# A new threshold reveals the uncertainty about the effect of school opening on diffusion of Covid-19

**DOI:** 10.1038/s41598-022-06540-w

**Published:** 2022-02-22

**Authors:** Alberto Gandolfi, Andrea Aspri, Elena Beretta, Khola Jamshad, Muyan Jiang

**Affiliations:** 1 Division of Science, New York University Abu Dhabi, Abu Dhabi, 129188 UAE; 2 Università di Pavia, Pavia, Italy

**Keywords:** Applied mathematics, Phase transitions and critical phenomena, Epidemiology, Mathematics and computing

## Abstract

Studies on the effects of school openings or closures during the Covid-19 pandemic seem to reach contrasting conclusions even in similar contexts. We aim at clarifying this controversy. A mathematical analysis of compartmental models with subpopulations has been conducted, starting from the SIR model, and progressively adding features modeling outbreaks or upsurge of variants, lockdowns, and vaccinations. We find that in all cases, the in-school transmission rates only affect the overall course of the pandemic above a certain context dependent threshold. We provide rigorous proofs and computations of the thresdhold through linearization. We then confirm our theoretical findings through simulations and the review of data-driven studies that exhibit an often unnoticed phase transition. Specific implications are: awareness about the threshold could inform choice of data collection, analysis and release, such as in-school transmission rates, and clarify the reason for divergent conclusions in similar studies; schools may remain open at any stage of the Covid-19 pandemic, including variants upsurge, given suitable containment rules; these rules would be extremely strict and hardly sustainable if only adults are vaccinated, making a compelling argument for vaccinating children whenever possible.

## Introduction

The question of keeping schools open or closing them has turned out to be one of the most debated issues of the Covid-19 pandemic. Schools have been closed at the early stages of the pandemic in almost every country, with classes held online for most of last year^1,2^; but the, sometimes hurriedly arranged, remote teaching has created great difficulties for more than one billion students, their teachers and communities^3,4^.

Many studies have been carried out trying to clarify the potential effects of school opening on the course of the Covid-19 outbreaks: they appear to be reaching different, and sometimes conflicting, conclusions. Several works^5–8^ find that closing schools has little impact on the number of cases, while others conclude that it is very effective^9–11^; a number of other studies determine that the influence of school opening on the course of the pandemic depends crucially on some implementation details, such as level of blending, use of masks etc.^12–18^. Even studies of the same situation reach opposite conclusions^19,20^. When trying to analyze available Covid-19 data using simulations of compartmental models, we observed a remarkable instability: the observed effect of school opening depended in a crucial way on small changes in the models parameters, and we kept oscillating between making the schools the culprits of the pandemic, or completely absolving them; see Fig. 3a–c below.

This situation raises the issue of understanding the mechanisms behind and the nature of a possible transition in the effect of school opening policies. Ideally, one would like to identify one or more parameters, measurable at least in some theoretical sense, summarizing the effects of school opening, with an explicitly, for as much as possible, known effect on the overall epidemiological indicators. A clearer understanding in this sense could inform data collection, analysis and release of information, and help providing guidelines for policy makers in concrete situations.

Our aim is to report of the identification of a mechanism which could explain the observed instability of effects and diversity of conclusions. To highlight the fundamental mechanisms at work, we started from the simplest compartmental models^21,22^ with two subpopulations, studying how changes in the transmission rate in one population affect the overall course of the infection. We first performed a theoretical study of the mathematical models, validating then the results using simulations with incrementally added more realistic features, and a comparative analysis of various data based studies.

The outcome of our analysis has been the existence of a perhaps surprising threshold, below which further reduction of in-school transmission, for instance by school closure, has a minimal effect, but above which school opening becomes the leading factor driving infections. We observe the transition in all scenarios, albeit with different vales of the threshold. Of particular interest is the case of a vaccination campaign of adults, in which the threshold for in-school transmission rates turns out to be extremely small, giving a strong indication of the need of a children vaccination campaign. The presence of a threshold for the in-school transmission rate can explain the divergent conclusions of several statistical studies, as they might have been observing the two opposite conditions. In addition, if in the situation under investigation school transmission is close to the threshold, epidemiological models, which necessarily rely on, often hard to estimate, parameters, might end up forecasting one or the other scenario depending on tiny changes in the calibration. Awareness of a threshold is crucial for modeling, data collection and analysis, and policies determination^23^.

Let us clarify, though, that, to focus on our main objective, we disregard many other relevant epidemiological issues concerning school opening, such as the possible role and availability of teachers^24^; the sustainability of school opening^25^; as well as psychological, cultural, educational aspects; that need then to be added when planning concrete policies.

In addition, variants, especially those which might be vaccine resistant, constitute a very challenging situation: from an abstract point of view, the situation is similar to that of an initial outbreak, as there are little awareness and immunization capabilities, so, broadly speaking, our first scenario below applies. On the other hand, the population has developed new individual and social responses, and therefore new assessment of various parameters of our investigation should be suitably modified. It is too early now to be able to make such adjustments, but the situation should be reevaluated should a resistant variant become prevalent.

## Results

### There is a phase transition in the effect of school transmission rates on the overall epidemic course during an outbreak (or a variant upsurge)

The effect of the in-school transmission rate $\beta _{11}$ on the course of the epidemic undergoes a phase transition with threshold

1 $$\begin{aligned} \beta _{11}^*= \beta _{22} S_2(0)/S_1(0). \end{aligned}$$

The total number of active cases is almost constant for all $\beta _{11} \in [0,\beta _{11}^*]$, and has a sharp increase for $\beta _{11} > \beta _{11}^*$. An effective containment of the effect of a change of $\beta _{11}$ is achieved if

2 $$\begin{aligned} \beta _{11} < \beta _{22} S_2(0)/S_1(0)- \alpha \sqrt{\beta _{12} \beta _{21} S_2(0)/S_1(0)}, \end{aligned}$$

while there is a substantial effect if

3 $$\begin{aligned} \beta _{11} > \beta _{22} S_2(0)/S_1(0)+ \alpha \sqrt{\beta _{12} \beta _{21} S_2(0)/S_1(0)}, \end{aligned}$$

where $\alpha$ is generally chosen to be a small integer (smaller than $\frac{\beta _{22} S_2(0)/S_1(0)}{\sqrt{\beta _{12} \beta _{21} S_2(0)/S_1(0)}}$ to make sense of (2)), analogous to the number of deviations away from a mean, used to describe a transition as plotted in Fig. 4. We take $\alpha =2$ in “Parameter calibration” section. Since in concrete cases, see “Parameter calibration” in "Methods" section, $\beta _{12}, \beta _{21}<<\beta _{22}$ the right hand sides of (2) and (3) are close to $\beta _{11}^*$.

Calculations are done in a linear approximation of the SIR model, which applies to the Covid-19 pandemic as the numbers of active cases are kept relatively low by containment measures in the early stages of the outbreaks^26^. In the linear approximation it is possible to formally compute the total number of cases up to a certain time ${\overline{t}}$, which corresponds to when a lock down is imposed. If the target is to contain the increase in the number of total cases up to ${\overline{t}}$ to a given percentage $\varepsilon$, an explicit formula allows to compute the maximal allowed value of $\beta _{11}$.

In a realistic example with total population and recovery rate $\gamma$ normalized to 1, setting $S_1(0)=0.2, S_2(0)\approx 0.8, \beta _{12}= \beta _{21} =0.5, \beta _{22}=2$, see “Parameter calibration” in "Methods" section, the critical point is $\beta _{11}^*\approx 8$. Assuming an initial fraction of $3 \times 10^{-5}$ of active cases in Subpopulation 2 and none in Subpopulation 1, a rescaled time frame of ${\overline{t}}=5$ (corresponding to approximately 50 days), and $\varepsilon =0.3$, a suitable value of $\alpha$ gives $\beta _{11} \le 6.344$.

**Figure 1:**
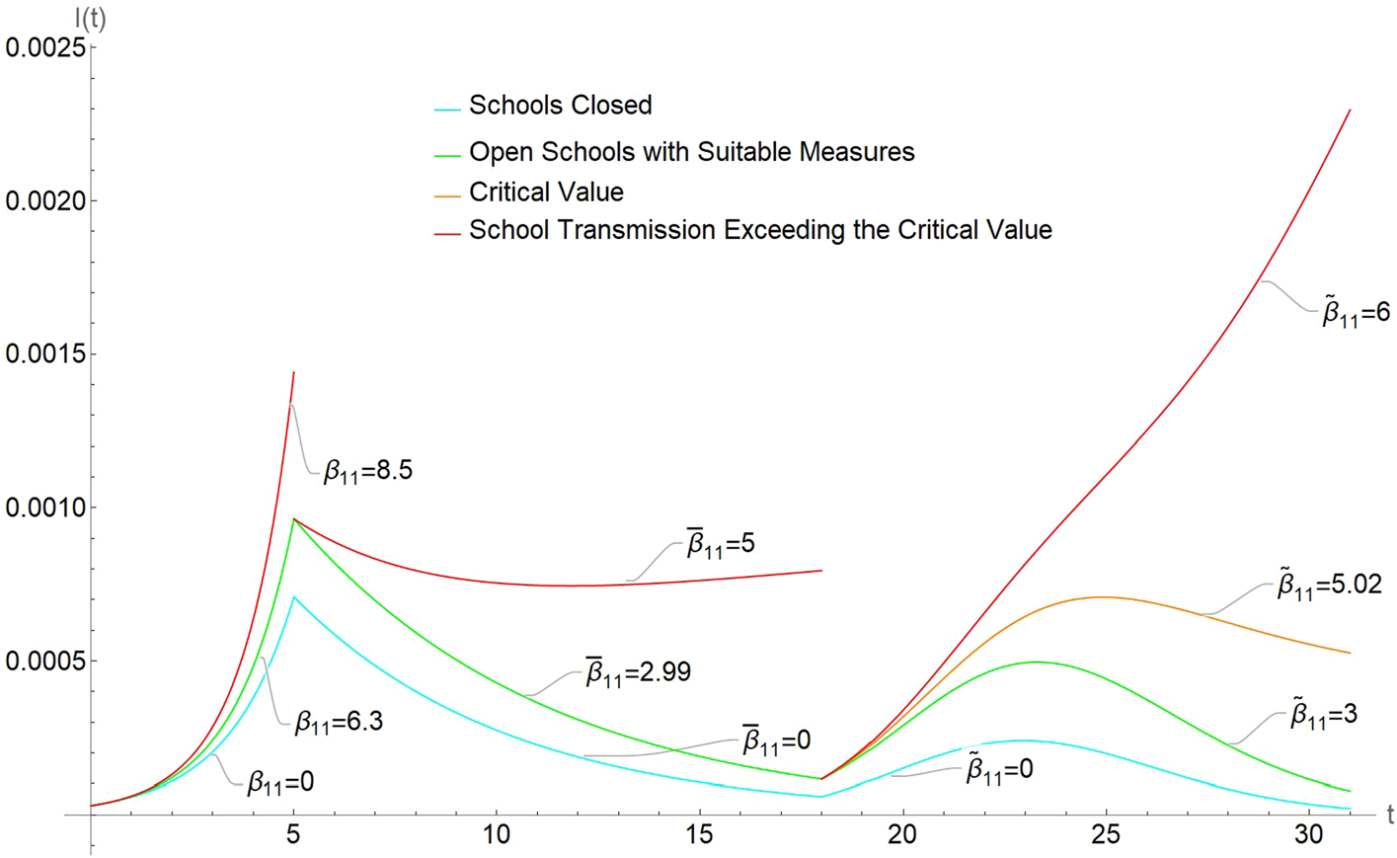
Daily active cases $I_1(t)+I_2(t)$ for various scenarios: outbreak or new strain upsurge, lockdown, and vaccination. In each case, there is critical value for the in-school transmission rate $\beta _{11}$. Cyan curve is with closed schools, green for safe opening, orange for critical values, red for values above criticality.

The first part of Fig. 1, for $t \in [0,5]$, shows a simulation of the active cases with the above values. One can see that school opening has a moderate effect for small values of $\beta _{11}$, and then the effect becomes dramatic as the values increase past the critical point.

It follows that closing schools, i.e. setting $\beta _{11}=0$, is of limited impact if the reproduction rate $\beta _{11} S_1(0)$ in school is somewhat lower than $\beta _{22} S_2(0)$, the external reproduction rate, and of substantial impact otherwise. This provides harmless school opening options, assuming that one has access to the reproduction rates in the subpopulations.

### The phase transition is preserved under lock-down, albeit with a different critical point

An analogous effect takes place when a lockdown is imposed. If at some time $\overline{t}$ transmission rates are reduced to values ${\overline{\beta }}_{ij}$, corresponding to a subcritical reproduction number, then the effect of ${\overline{\beta }}_{11}$ on the total number of active cases undergoes the same phase transition as during the outbreak, but with critical point

4 $$\begin{aligned} {\overline{\beta }}_{11}^{*}=\frac{1}{S_{1}({\overline{t}})} -\frac{{\overline{\beta }}_{12} {\overline{\beta }}_{21} S_{1}({\overline{t}}) S_{2}({\overline{t}})}{ S_{1}({\overline{t}}) (1 - {\overline{\beta }}_{22} S_{2}({\overline{t}}))}. \end{aligned}$$

More precisely, let

$$\begin{aligned} A=\frac{\Delta S ({\overline{\beta }}_{11})}{(S_1({\overline{t}})+ S_2({\overline{t}}))} =\frac{(S_1(\infty ) -S_1({\overline{t}})+ S_2(\infty )- S_2({\overline{t}}))}{(S_1({\overline{t}})+ S_2(\overline{t}))} \end{aligned}$$

indicate the attack rate of the epidemic, i.e. the fraction of the initially susceptible population that is eventually infected by the disease in the course of the epidemic from ${\overline{t}}$ to complete eradication. It turns out that a sufficient condition to ensure that $\Delta S ({\overline{\beta }}_{11})$ does not exceeds $(1+\varepsilon )\Delta S (0)$ is

5 $$\begin{aligned} {\overline{\beta }}_{11}< F(\varepsilon ) \end{aligned}$$

where *F* (see (26) below) is a function that depends on the proportions of active cases and susceptible individuals at time ${\overline{t}}$.

In a realistic example continuing the one for the outbreak, with ${\overline{\beta }}_{12}= {\overline{\beta }}_{21} =0.25, {\overline{\beta }}_{22}=1,$ we get $\overline{\beta }_{11}^{*}\approx 4.7630$. In addition, we take $\varepsilon =0.3$, see “Linear approximation during the initial phase of an outbreak or new strain upsurge” in "Methods" section; in order to contain the increase in attack rate to no more than 30% one needs now to have

$$\begin{aligned} {\overline{\beta }}_{11} < 2.9944. \end{aligned}$$

Although in a different scenario, this is smaller than the value 6.344 found in the outbreak, as there the aim was just to avoid producing an even more extended diffusion of the infection.

The second part of Fig. 1, for $t \in [5,18]$, illustrates active cases in the lockdown scenario, with the above values of the model parameters.

When considering a complete outbreak-lockdown cycle, the attack rate undergoes a similar transition, depending on the values of the two transmission rates $\beta _{11}$ and ${\overline{\beta }}_{11}$. If the pair is sufficiently closed to (0, 0), then there is little change in $\Delta (S)$, while there is a drastic change for larger values of the two transmission rates (see Fig. 7).

### Success of widespread vaccination of non-schooling individuals requires internal reproduction number in schools to be subcritical

If a vaccination campaign for not-in-school individuals is carried out, the total number of cases from a restart of the epidemic to the complete disappearance due to vaccination undergoes an analogous phase transition, with threshold

6 $$\begin{aligned} {\widetilde{\beta }}_{11}^{*}= 1/{S_1(0)}. \end{aligned}$$

The attack rate is only moderately changed for $\widetilde{\beta }_{11}$ below the threshold, while the outcome of the vaccination process is substantially disrupted for larger values of ${\widetilde{\beta }}_{11}$.

Notice that if ${\widetilde{\beta }}_{11}<{\widetilde{\beta }}_{11}^{*}$ then the in-school reproduction number is $R_S={\widetilde{\beta }}_{11} S_1(0) < 1$ i.e. $R_S$ is subcritical.

In a realistic case, continuing with the data from the example above, we now suppose a vaccination program is introduced targeted to a 60% coverage in about 3 months (Israel kept this pace at the time we are writing, with schools almost completely closed). In Fig. 1, this corresponds to $t\in [18,31]$, and now $S_1(0) = 0.198872$ is the susceptible at the start of this simulation, which is equal to the susceptible population calculated for the end of the previous simulation for lockdown (corresponding to t = 18 in Fig. 1). Then $R_S=0.198872 \times \widetilde{\beta }_{11}$, and the critical value for ${\widetilde{\beta }}_{11}$ is 5.02836. To achieve a sensible containment that limits the number of extra infections to no more than 30% one needs to take $\widetilde{\beta }_{11}<3.03111$. See “Simulations of the phase transition during vaccination” in "Methods" section for further details on this simulation.

### From the point of view of containing the epidemic, schools can be kept open at all times, with strict control measures

Taken together, the results obtained from simple SIR models with subpopulations show that, although the values of the critical points are different, opening of schools would not seriously affect the course of the pandemic at all times, provided the internal transmission rate is kept low enough. On the other hand, if the control is released, then the effect of school opening becomes dramatic.

Figure 1 summarizes the numbers of active cases in the three scenarios we have analyzed: the cyan curve corresponds to closed schools, while the green one is a subcritical pattern; the red curves, instead, show the risk that the pandemic spirals out of control because of insufficiently controlled school opening.

Notice that the explicit values that we give in Formulas (1), (4) and (6) are relevant from the theoretical point of view, as they indicate that the critical thresholds are different. Their specific values, however, could be hard to estimate from these formulas. The initial fraction of infected in (1), for instance, is almost impossible to estimate, due to the initial absence of awareness and testing. Other methods and more details models would be needed for a careful estimation of the threshold in concrete cases.

An analogous behavior takes place in more elaborate and realistic models, involving presymptomatic, asymptomatic, testing, isolation etc. Critical values appear for the in-school transmission rate, below which the effect of school opening on the epidemic trajectory is extremely contained. We provide simulations in “A SPIAR model” in section (see Fig. 10), and evidence from case studies here below.

## Discussion

### Evidence of phase transition appears in several data driven case studies

The effect of a phase transition seems to appear in all data driven studies (see “Other case studies” in section). Most studies reach a definite conclusion: in some cases, the data or the model after calibration correspond to a subcritical regime, so that the study ends up asserting the almost irrelevance of school opening on the pattern of the epidemic for all the analyzed cases; in other cases, the study determines a supercritical setting, and then comes to the opposite conclusion.

**Figure 2:**
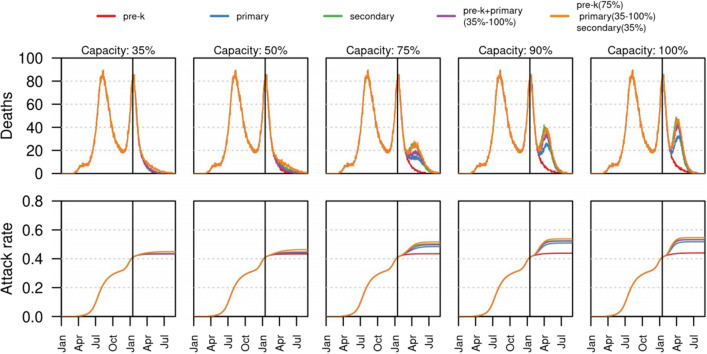
The impact of school reopening strategies in time as simulated in^17^ from data from Bogotà, Colombia, for various values of the capacity, i.e. the percentage of students allowed back at school. Each column shows a different capacity level. Top panel shows the median daily incidence of deaths for each reopening strategy based on grades. Bottom panel shows the estimated attack rate for each of the reopening scenarios. Vertical black line shows the timing of school reopening (January 25, 2021). All scenarios were simulated up to August 31, 2021.

Some works include one or more parameters that can be modulated to envision the effect of school reopening. In these cases, one can see the effect of a sharp transition from a subcritical, acceptable reopening, to an excessively impactful one. In España et al., Figs. 4 and 5, for instance, one can see that up to 50% capacity the effect of opening schools is almost negligible, while it becomes substantial above 75%; this is a likely indication of a critical point between these values^17^. For convenience, their Fig. 4 is reproduced here in Fig. 2.

A very detailed study of school opening in The Netherlands is conducted in Rozhnova et al. , and their conclusions are a clear description of the phase transition^12^. Using a data driven, elaborate model, Rozhnova et al. claim that their “analyses suggest that the impact of measures reducing school-based contacts depends on the remaining opportunities to reduce non-school-based contacts^12^. If opportunities to reduce the effective reproduction number ($R_e$) with non-school-based measures are exhausted or undesired and $R_e$ is still close to 1, the additional benefit of school-based measures may be considerable, particularly among older school children.” The first scenario of Rozhnova et al.  corresponds to a subcritical in-school transmission rate, so that the effect of closing schools would be very moderate. The second scenario seems to correspond to an in-school transmission rate around the critical value, so that both containment, in- or out-of-school, are effective^12^﻿.

Yuan et al. uses a detailed compartmental model and data from the second semester 2020 in Toronto, an outbreak context, to estimate the likely impact of school opening; with parameters estimated and collected from literature, the paper finds that opening school has little effect on the overall course of the pandemic: in all scenarios presented in their Figs. 2 and 4 the difference between school opening and closure is extremely contained^16^. The findings of the research is then consistent with our phase transition scenario. In particular Yuan et al., find that “school reopening was not the key driver in virus resurgence, but rather it was community spread that determined the outbreak trajectory”; in other words, the parameters of the models, although not explicitly given in the paper, are such that the external transmission is preponderant^16^. As an additional finding, it is observed in Yuan et al. that, according to their model, “brief school closures did reduce infections when transmission risk within the home was low”^16^: in this case, a reduced transmission rate outside makes the one in school likely supercritical.

### The role of phase transitions

Phase transitions, like the one occurring at $R_0=1$ or the one we detect for in-schoool transmission rates, are fundamental in epidemiology^23^. They consist of the fact that changes in one parameter, the in-school transmission rate in our case, produce almost no effects except when the threshold is crossed; at that point, however, a small change in the parameter determines completely different behaviors.

The presence of a threshold for the in-school transmission rate can explain the divergent conclusions of data driven studies, as they might have been observing two different phases. The presence of a phase transition can also dramatically disrupt forecasts based on calibrated compartmental models: one calibration might lead to a subcritical phase, in which the transmission in school is irrelevant, and another, based on possibly similar data, might lead to a supercritical phase, in which in-school transmission is the driving factor of the pandemic. This phenomenon is known to affect epidemic forecasts^27^, and we think it might be the reason beyond the mentioned conflicting conclusions of several studies.

To make things worse, even retrospective studies trying to evaluate the role of school openings or closures on the evolution of the pandemic run the risk of being completely untrustworthy. Covid-19 data are affected by enormous errors, due to the presence of asymptomatic, lack and partial reliability of testing, difficulty in assessing close contacts etc. It follows that estimation of parameters for both statistical and model based studies are affected by large errors. In the presence of a threshold, even small errors can lead to incorrect attribution of the situation under observation to one phase, or to conflicting attributions to two opposite phases by different studies. In such scenario, a retrospective study could misclassify the effect of school opening or closure; and different studies even based on almost the same data might end up reaching opposite conclusions. We explicitly illustrate this phenomenon with a simulation in the next section.

Finally, awareness about the presence of a phase transition suggests the type of measurements that could be carried out, analyzed and finally released to the public. In our case, for instance, one could consider adapting our model to specific local situations, and then measuring in-school transmission rates; these can then be used as basis for local policies about school opening, and also as a possible public indicator of the potential risk of interventions on schools. The information that the in-school transmission rate is approaching a critical level would certainly stimulate and justify the reinforcement of containment measures. The findings about vaccination strongly support vaccinating children as well.

### Confounding effects on retrospective studies

**Figure 3:**
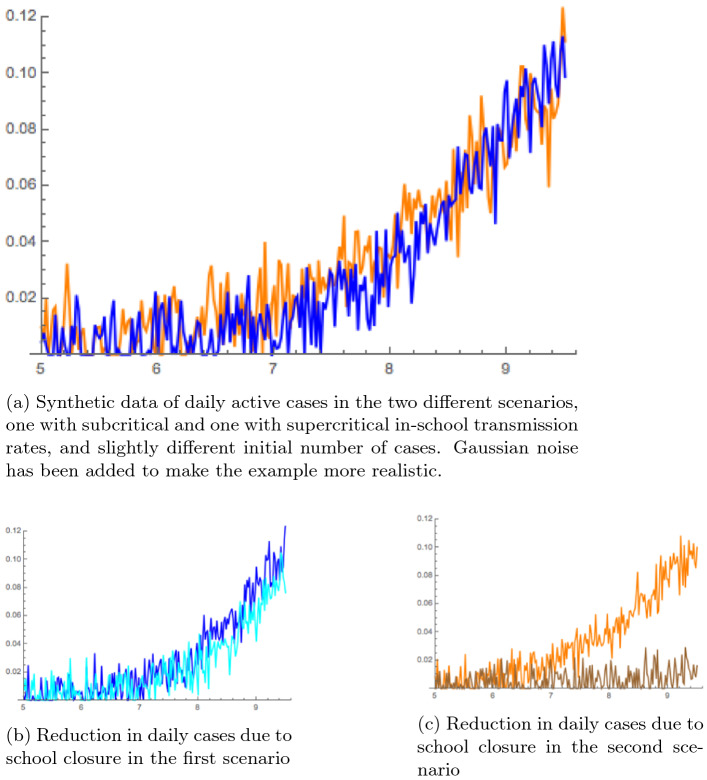
Active cases in two different scenarios.

**Figure 4:**
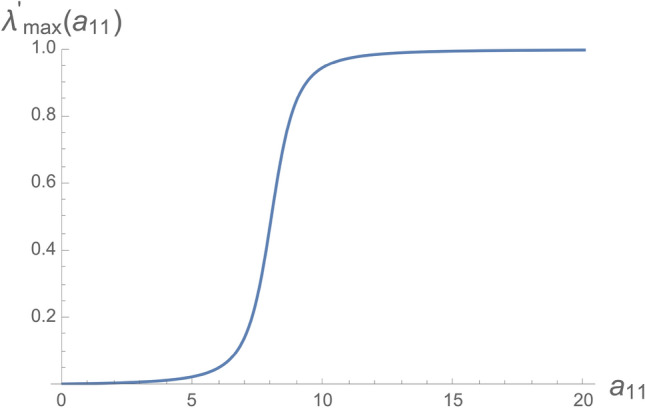
Derivative of the largest eigenvalue of $A=(a_{i j})$ with respect to $a_{11}$, with $a_{12} = a_{21} = 0.5$ and $a_{22} = 8$.

**Figure 5:**
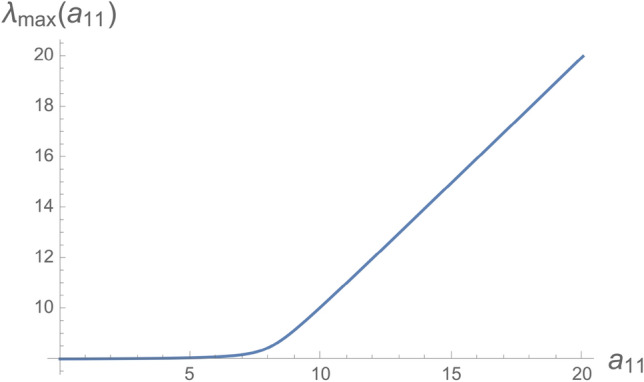
The largest eigenvalue of $A=(a_{i j})$ as function of $a_{11}$, with $a_{12} = a_{21} = 0.5$ and $a_{22} = 8$.

**Figure 7:**
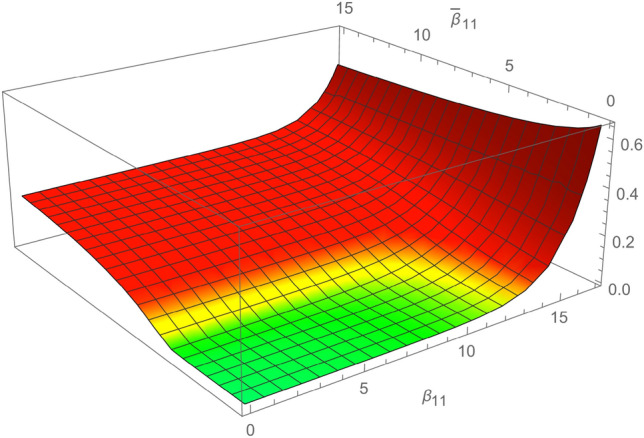
Total number of cases at resolution of outbreak for varying values of $\beta _{11}$ and ${\overline{\beta }}_{11}$.

**Figure 10:**
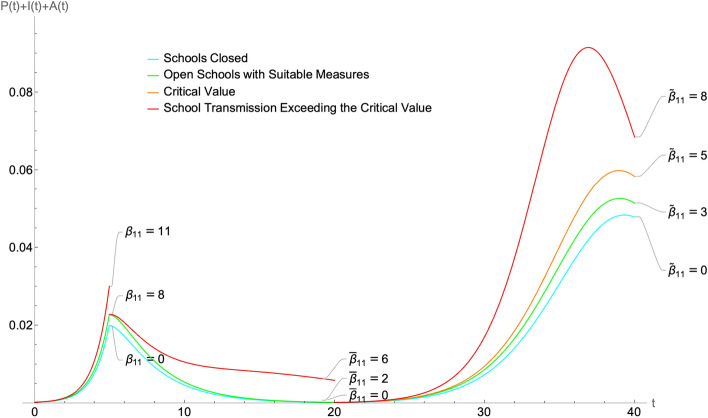
Daily cases for the three scenarios of outbreak, lockdown, and vaccination in an SPIAR model. In each case, there is critical values for the in-school transmission rate $\beta _{11}$, as for SIR model. Cyan curve is with closed schools, green for safe opening, orange for critical values, red for values above criticality.

In the noisy, synthetic data in Fig. 3a, the number of daily infected in a population have been generated with the same parameters, except that

7 $$\begin{aligned} \text { first scenario: }&\beta _{11} = 10, \beta _{2,2}=2, I_2(0)= 3 \times 10^{-5} \end{aligned}$$

8 $$\begin{aligned} \text { second scenario: }&\beta _{11}= 6, \beta _{2,2}=2.57, I_2(0)= 5.5 \times 10^{-6}. \end{aligned}$$

In school transmission rates are supercritical in the first case, and subcritical in the second. But the different number of initial cases, a value that is subject to errors of various order of magnitudes and is quite arbitrarily assigned in the various studies, makes the two trajectory basically indistinguishable.

In a retrospective study one is forced to assign an initial value to the number of infected, and then estimate other parameters from the observations. Both scenarios are then plausible, depending on the chosen initial values. As Fig. 3b confirms, the research would conclude in the first scenario, that closing schools would have been basically useless. In the second scenario, however, the opposite conclusion would be drawn, as illustrated by Fig. 3c.

## Conclusions

We have identified the presence of different phases for the effect of the in-school transmission rates on the course of a Covid-19 like epidemic.

Such results provide evidence in favor of keeping the schools open when specific epidemiological conditions are met and preventive measures are respected: the key condition is that the transmission rate in schools must be kept below a certain threshold that depends on the situation. As the threshold might not be easily determinable nor achievable, however, there can be contingent motives for school closure if policy makers, as it happened in most locations during the Covid-19 pandemic, are not aware or able to exploit the critical threshold. Awareness about the critical threshold can in any case suggest directions for the analysis of locally adapted models, data collection and exploration, and public release, and can give policy makers sound instruments for containing school closures.

In addition, the presence of a threshold is the likely cause of the opposing views that many studies have presented, some asserting almost irrelevance of school opening, and others pointing to its significant effects. Minimal changes in the overall conditions, or in values of the estimated parameters may determine one phase of the other; this may result in different attributions of responsibility to school opening, and creates the possibility of an arbitrary identification of the phase due to parameter estimation in the presence of very noisy data.

Finally, we have seen that with a vaccination campaign being carried out largely for out-of-school individuals only, there is a threshold below which schools can still be opened; it corresponds, however, to an internal reproduction number that would eradicate the virus if schools were completely isolated. As measure to achieve such a low reproduction number are highly demanding, this level of containment seems to be sustainable for brief periods only, after which vaccination for children becomes the only viable possibility to return to normality.

## Limitations, related and future works

Although the presence of a phase transition in the effect of the school transmission rate in the overall course of the epidemic seems to have been unnoticed so far in the literature, there are many works related to ours.

For a different perspective, focused on the sustainability of opening from the point of containing the number of cases of a single school, one can see^25^.

Compartmental models with two subpopulations are discussed in many works in general terms^28^; and then applied to the school opening issue in data driven analyses^7,11,16,17^: we discuss the relation of some of these results with our work in “Other case studies” in "Methods" section.

Finally, other papers^6–8^ make a purely statistical evaluation of the effect of school opening (see “Other case studies” in "Methods" section).

Our work has several limitations. Our results are based on abstract, simplified models, and, although they seem to be stable when more detailed features are included, we cannot ensure that they always take place in more complex models.

Even when a critical value can be estimated, ensuring that the transmission rates are below their relative thresholds is clearly a matter of distancing, testing, and other measures^29^. We do not elaborate here on how to develop a set of possible interventions, and on how to measure their success in containing the transmission rates in schools.

There are several directions for future work and research.

From the practical point of view, our analysis needs to be adapted to local and contingent situations adding specific details to the model, and collecting and analyzing appropriate data before becoming a viable tool for policy makers.

From the mathematical point of view, it would be interesting to ascertain the presence and behavior of the phase transition in the non linear models. Also, it would be relevant to explore the analogous phenomenon when the cross terms are not small with respect to those in the main diagonal, a situation which could explain the dynamics of vaccinated vs. unvaccinated population. Finally, one could evaluate the presence of similar phenomena with more than two subpopulations, in order to detect which combinations drive the pandemic, and which internal transmissions can be disregarded up to a certain threshold.

## Figures and Tables

**Figure 6:**
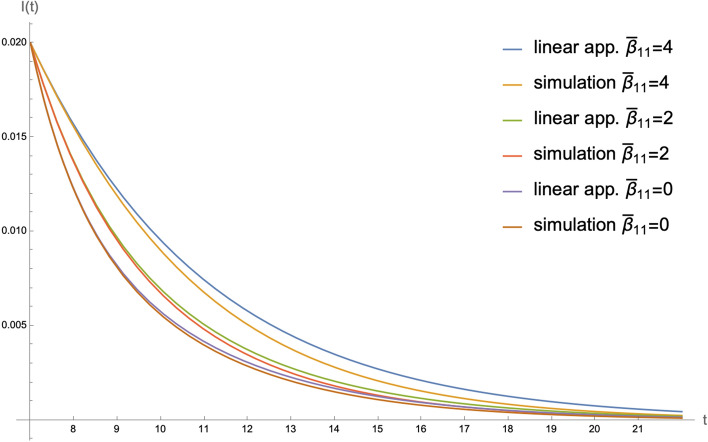
Effectiveness of the linear approximation of the SIR model for lockdown; the figure shows the total active cases numerically simulated with realistic parameters and varying ${\overline{\beta }}_{11}$: in each test, simulations from the differential system and from its linear approximation are indistinguishable.

**Figure 8:**
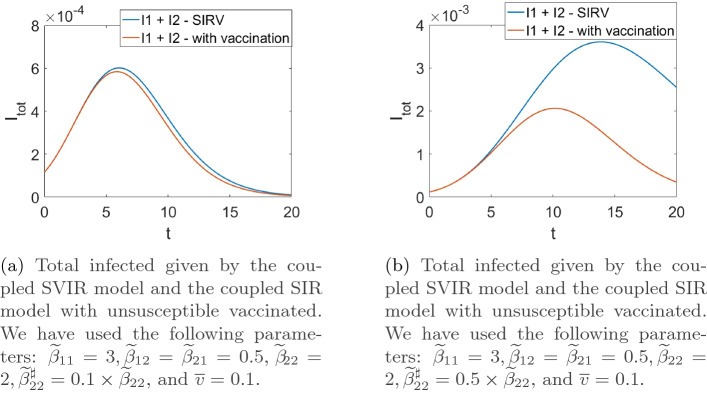
Comparison of the coupled SVIR model with the coupled SIR model with unsusceptible vaccinated. Initial conditions are those reported in “Simulations of the phase transition during vaccination” section.

**Figure 9:**
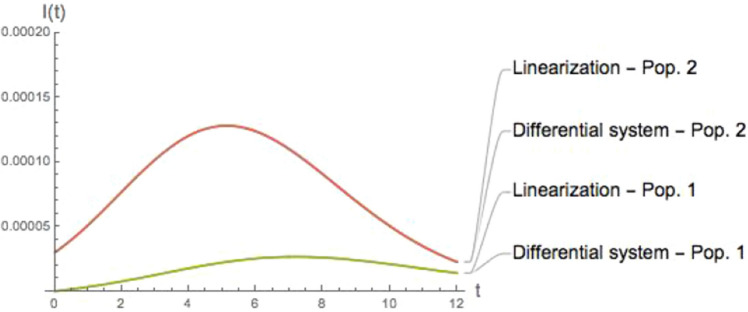
Effectiveness of the linear approximation of the SIR model with vaccination; the figure shows the total active cases numerically simulated with realistic parameters, $\widetilde{\beta }_{11}=3,\widetilde{\beta }_{22}=2$: simulations from the differential system and from its linear approximation are indistinguishable. Total active cases are small because the vaccinated period is assumed to start at the end of the lock-down period.

## Methods

### Compartmental models

In order to evaluate the effect of school opening on the course of the epidemic we use compartmental models, as they proved capable of predicting the courses of outbreaks in many instances^30^. We start with the simplest SIR model with unit total population, and two subpopulations $i= 1, 2$ where Subpopulation 1 refers to students (in-school) while Subpopulation 2 includes the remaining population including teachers and staff. The size of Subpopulation *i* is $n_i$; we indicate by $S_i, I_i, R_i$ the susceptible, infected, and recovered individuals, respectively, within Subpopulation *i*. By definition, Subpopulations 1 and 2 are complementary, so we avoid any double counting. The transmission rate from Subpopulation *j* to *i* is indicated by $\beta _{ij}$. More features are added later on.

We make a sequence of theoretical claims concerning the effect of the contact rate in the subclass representing schools. Most of the claims are verified in suitable linear approximations of the SIR model; each result is then complemented with numerical simulations.

The linear approximations give very close approximations of the non linear model as in the entire course of the current COVID-19 pandemic the proportion of active cases $I=I_1+I_2$ is kept relatively low by either containment measures, lockdowns, or vaccinations: until the time of writing this work the average, taken from publicly available data, of the maximum number of active cases in the most exposed countries is around 1%, with an SD of about 0.8%. These values are compatible with our assumed hypothesis, and numbers were much lower for most of the time in most countries.

### SIR model with two subclasses

We first consider the simplest model of interest, represented in terms of a coupled SIR system

9 $$\begin{aligned} {\left\{ \begin{array}{ll} S_1'=-\beta _{11}S_1I_1-\beta _{12}S_1I_2\\ I_1'=\beta _{11}S_1I_1+\beta _{12}S_1I_2-I_1\\ R_1'=I_1\\ S_2'=-\beta _{21}S_2I_1-\beta _{22}S_2I_2\\ I_2'=\beta _{21}S_2I_1+\beta _{22}S_2I_2-I_2\\ R_2'=I_2 \end{array}\right. } \end{aligned}$$

Notice that the recovery rate is the same in the two subpopulations as for COVID-19 they seem to depend on the severity of the infection but not directly on age^31–33^, and time is rescaled so that it is equal to 1. This makes time unit of about 10–14 days^34^. In addition, $\beta _{12}, \beta _{21}$ are generally smaller than $\beta _{11}, \beta _{22}$^28,35^. We intend to compare the attack rates $\Delta S (\beta _{11})= S_1(t_a)-S_1(t_b)+ S_2(t_a)-S_2(t_b)$ between two suitable times $t_a<t_b$, as function of the in-school transmission rate $\beta _{11}$; here $\beta _{11}= 0$ corresponds to schools being closed, and $\beta _{11}> 0$ corresponds to schools being open with varying degrees of physical distancing and other containment measures in place.

### Parameter calibration

Alongside the rigorous proofs for the linearized models, we perform several simulations with realistic parameters, which are calibrated as follows.

Time is rescaled so that $\gamma =1$, a unit being approximately 10 days.

To calibrate transmission rates $\beta _{i,j}$, we start from the ratio of contact rates as can be extracted from Prem et al.^35^. This is a pre Covid-19 accurate study of contact rates, and we assume that the ratios of contact rates has remained approximately the same during the pandemic, with absolute values modified by awareness and measures. There are no equivalent studies for the pandemic period, and the values identified in some local studies^28,36^ are in agreement with what we find.

According to this scheme, the cross transmissions rates $\beta _{12}$ and $\beta _{21}$ are calibrated in relation to $\beta _{22}$; Soyoung et al. estimate $\beta _{12}/\beta _{22} \approx 7/47$ in the early times of the pandemic^28^. A slightly larger value of this ratio is obtained by considering the typical social contacts^35,36^, in which the cross contacts are about 1/2 of the contacts among adults, and the reduced susceptibility of children is estimated to be about 1/2 that of adults^37^: this gives $\beta _{12}/\beta _{22} \approx 1/4$. As our considerations work better with $\beta _{12}/\beta _{22}$ small, we use the last more conservative estimate. For $\beta _{11}$, it can be extracted from Prem et al.^35^ that $\beta _{11} \approx 6 \beta _{22}$  can be taken as first reference value, to be later varied according to school opening policies.

Using the reproduction matrix *A* in (12) below, one can write its largest eigenvalue in terms of $\beta _{22}$. Since the largest eigenvalue equals $R_t$, the overall reproduction number of the pandemic, one can use the estimated values of $R_t$ to get an evaluation of $\beta _{22}$. With the reference values above, it turns out that in fact $\beta _{22} \approx R_t$; this is another indication of the phase transition phenomenon, as for the above reference values the school transmission rate is almost irrelevant. For the outbreak and vaccination scenarios we take $\beta _{22} \approx R_t \approx 2$, and for the lockdown $\beta _{22} \approx R_t \approx 1$.

### Linear approximation during the initial phase of an outbreak or new strain upsurge

A suitable linear approximation for the initial period of the first outbreak, or of any of the possible infection waves taking place after a successful lockdown, is the following

10 $$\begin{aligned} {\left\{ \begin{array}{ll} S_1'=-\beta _{11}S_1(0)I_1-\beta _{12}S_1(0)I_2\\ I_1'=(\beta _{11}S_1(0)-1)I_1+\beta _{12}S_1(0)I_2\\ S_2'=-\beta _{21}S_2(0)I_1-\beta _{22}S_2(0)I_2\\ I_2'=\beta _{21}S_2(0)I_1+(\beta _{22}S_2(0)-1)I_2\\ \end{array}\right. } \end{aligned}$$

from which we extract the second and the fourth equations for $I_1, I_2$. In vector form we have

11 $$\begin{aligned} \vec {I}'=(A- \text {Id})\vec {I} \end{aligned}$$

where $\text {Id}$ is the $2\times 2$ identity matrix and

12 $$\begin{aligned} A= \begin{bmatrix} a_{11} &{} a_{12}\\ a_{21} &{} a_{22} \end{bmatrix} := \begin{bmatrix} \beta _{11}S_1(0) &{} \beta _{12}S_1(0)\\ \beta _{21}S_2(0) &{} \beta _{22}S_2(0) \end{bmatrix} \end{aligned}$$

is the reproduction matrix^38^.

#### Lemma A.1

The solution of (11) is

13 $$\begin{aligned} \vec {I}=e^{-t}e^{At}\vec {I}_0 =e^{-t}(e^{\lambda _{\text {max}}t}\vec {W}+e^{\lambda _{\text {min}}t}\vec {V}) \end{aligned}$$

where $\lambda _{\text {max}},\lambda _{\text {min}}$ are the positive eigenvalues of the matrix *A*, and $\vec {W}>0$.

The proof is in Appendix A.

The largest eigenvalue of *A* is the overall reproduction number $R_0$^38^, and the early evolution of the epidemics depends on the size of $\lambda _{\text {max}}$, and on the spectral gap $\lambda _{\text {max}}- \lambda _{\text {min}}$. This is the so-called slaved phase, in which the active cases of both populations are both lead by approximately the same exponential growth^38^.

### Dependence of the largest eigenvalue of $2\times 2$ matrices from the first entry

To get a first indication of a sudden change in the effect of the in-school transmission rate $\beta _{11}$, we study the behavior of the largest eigenvalue of a quite general $2\times 2$ matrix as function of its first entry. Let

$$\begin{aligned} \lambda '(a_{11}):= \frac{d\lambda _{max}}{d a_{11}} \end{aligned}$$

then the following estimate holds:

#### Theorem A.2

Let $A=(a_{i j})$ be a $2\times 2$ matrix with positive entries and let $\lambda _{max}(a_{11})>\lambda _{min}(a_{11})$ be its eigenvalues. We have that for any $\alpha \in \big (0,\frac{a_{22}}{\sqrt{a_{21}a_{12}}}\big )$

14 $$\frac{{\lambda _{{max}}^{{\prime }} (a_{{22}} + \alpha \sqrt {a_{{12}} a_{{21}} } ) - \lambda _{{max}}^{{\prime }} (a_{{22}} - \alpha \sqrt {a_{{12}} a_{{21}} } )}}{{\lambda _{{max}}^{{\prime }} (a_{{22}} - \alpha \sqrt {a_{{12}} a_{{21}} } ) - \lambda _{{max}}^{{\prime }} (0)}} = :\frac{{\Delta _{1} }}{{\Delta _{0} }} = \frac{{\Delta _{1} }}{{\Delta _{2} }} := \frac{{\lambda _{{max}}^{{\prime }} (a_{{22}} + \alpha \sqrt {a_{{12}} a_{{21}} } ) - \lambda _{{max}}^{{\prime }} (a_{{22}} - \alpha \sqrt {a_{{12}} a_{{21}} } )}}{{\lambda _{{max}}^{{\prime }} (2a_{{22}} ) - \lambda _{{max}}^{{\prime }} (a_{{22}} + \alpha \sqrt {a_{{12}} a_{{21}} } )}} \ge \frac{{2\alpha }}{{\sqrt {4 + \alpha ^{2} } - \alpha }}$$

The proof is in Appendix B. Figure 4 shows an example of the change in derivatives.

As a consequence, if, for instance, $\alpha =2$ as we will assume from now on, then

$$\begin{aligned} \Delta _1\ge 4.8 \Delta _0=4.8 \Delta _2. \end{aligned}$$

Simple calculations in Appendix D show then that if $0<a_{11}\le a_{22}-\alpha \sqrt{a_{12}a_{21}}$, which is realistic since $a_{22}^2>>a_{12}a_{21}$,

$$\begin{aligned} \lambda _{max}(2a_{22})-\lambda _{max}(0)\ge 4.8(\lambda _{max}(a_{11})-\lambda _{max}(0)). \end{aligned}$$

The change in the largest eigenvalue is illustrated in Fig. 5.

### A phase transition for school opening during an outbreak

We now want to see a similar behavior in the active cases $I_1+I_2$ in the coupled SIR model (9). Let

$$\begin{aligned} \begin{aligned} \lambda _{\text {max}}(0)&=\lambda _0,\qquad \lambda _{\text {min}}(0)&=\widetilde{\lambda _0} \\ \lambda _{\text {max}}(a_{22}-\alpha \sqrt{a_{12}a_{21}})&=\lambda _1,\qquad \lambda _{\text {min}}(a_{22}-\alpha \sqrt{a_{12}a_{21}})&=\widetilde{\lambda _1}\\ \lambda _{\text {max}}(a_{22}+\alpha \sqrt{a_{12}a_{21}})&=\lambda _2,\qquad \lambda _{\text {min}}(a_{22}+\alpha \sqrt{a_{12}a_{21}})&=\widetilde{\lambda _2}. \end{aligned} \end{aligned}$$

and denote by $\vec {W}^i=(W_1^i,W_2^i)$ and $\vec {V}^i=(V_1^i,V_2^i)$, for $i=0,1,2$, the vectors $\vec {W}$ and $\vec {V}$ in (13) corresponding to $\lambda _0, \lambda _1, \lambda _2$, and $\widetilde{\lambda _0}, \widetilde{\lambda _1}, \widetilde{\lambda _2}$, respectively. Note that, without loss of generality, we can assume that $V^i_1>0$, for $i=0,1,2$.

Let $\vec {I}^{\lambda _j}(t), j=0,1,2$ denote the infected at time *t* corresponding to eigenvalues $\lambda _j,j=0,1,2$ respectively.

#### Corollary A.3

For all $k>0$, there exists $T_k>0$ such that for all $t\ge T_k$

$$\begin{aligned} |\vec {I}^{\lambda _2}(t)-\vec {I}^{\lambda _0}(t)|\ge k|\vec {I}^{\lambda _1}(t)-\vec {I}^{\lambda _0}(t)|. \end{aligned}$$

The proof is in Appendix C. This identifies $a_{22}$ as the critical point for the effect of the coefficient $a_{11}$ on the largest eigenvalue of *A*; by the relations in (12), the critical point for the effect of $\beta _{11}$ on the overall pandemic is then $\beta _{11}^*= \beta _{22} S_2(0)/S_1(0)$.

### Containment of the effect of school opening during an outbreak

For $i=1,2$ and some ${\overline{t}}>0$, let

15 $$\begin{aligned} \mathcal {I}_i= \int _{0}^{{\overline{t}}} I_i(\tau ) d\tau . \end{aligned}$$

Integrating the first and third equations of (10), we get

16 $$\begin{aligned} \Delta S_i= & {} S_i(0)-S_i({\overline{t}}) = -\int _{0}^{{\overline{t}}} S_i'(\tau ) d\tau \nonumber \\= & {} \int _{0}^{{\overline{t}}} (I_i'(\tau )+ I_i(\tau ))d\tau \nonumber \\= & {} I_i({\overline{t}})-I_i(0)+\mathcal {I}_i. \end{aligned}$$

On the other hand, integrating the second and fourth equations of (10) in $[0,{\overline{t}}]$, we get

17 $$\begin{aligned} {\left\{ \begin{array}{ll} I_{1}({\overline{t}})-I_1(0)=(\beta _{11}S_1(0)-1)\mathcal {I}_1+\beta _{12}S_1(0)\mathcal {I}_2\\ I_{2}({\overline{t}})-I_2(0)=\beta _{21}S_2(0) \mathcal {I}_1+(\beta _{22}S_2(0) -1)\mathcal {I}_2, \end{array}\right. } \end{aligned}$$

whose solution is

18 $$\begin{aligned} {\left\{ \begin{array}{ll} \mathcal {I}_1=-\frac{I_1(0)-I_{1}({\overline{t}}) + \beta _{12} (I_2(0)-I_{2}({\overline{t}})) S_{1}(0) - \beta _{22} (I_1(0)-I_{1}({\overline{t}})) S_{2}(0) }{-1+\beta _{11} S_{1}(0) (1 - \beta _{22} S_{2}(0)) + \beta _{22} S_{2}(0) + \beta _{12} \beta _{21} S_{1}(0) S_{2}(0)}\\ \mathcal {I}_2=-\frac{ I_2(0)-I_{2}({\overline{t}}) - \beta _{11}( I_2(0)-I_{2}({\overline{t}})) S_{1}(0) + \beta _{21} (I_1(0)-I_{1}({\overline{t}})) S_{2}(0) }{-1+\beta _{11} S_{1}(0) (1 - \beta _{22} S_{2}(0)) + \beta _{22} S_{2}(0) + \beta _{12} \beta _{21} S_{1}(0) S_{2}(0)}. \end{array}\right. } \end{aligned}$$

The attack rate is then

19 $$\begin{aligned} A(\beta _{11}):= & {} \frac{1}{S_1(0)+S_2(0)}(\Delta S_1+\Delta S_2)\nonumber \\= & {} \frac{1}{S_1(0)+S_2(0)}(I_{1}(\overline{t})-I_1(0)+\mathcal {I}_1+I_{2}({\overline{t}})-I_2(0)+\mathcal {I}_2) \nonumber \\= & {} \frac{1}{S_1(0)+S_2(0)}\Big \{\big (\beta _{11}\beta _{22}S_1(0)S_2(0) \nonumber \\&\quad -\beta _{12}\beta _{21}S_1(0)S_2(0)\big )\big [I_1(\overline{t})-I_1(0)+I_2(\overline{t})-I_2(0)\big ]\nonumber \\&\quad +\big (\beta _{21}S_2(0)+\beta _{11}S_1(0)\big )\big [I_1(0)-I_1(\overline{t})\big ]\nonumber \\&\quad +\big (\beta _{12}S_1(0)+\beta _{22}S_2(0)\big )\big [I_2(0)-I_2(\overline{t})\big ]\Big \}\nonumber \\&\quad \times \Big \{1-\beta _{11} S_{1}(0) (1 - \beta _{22} S_{2}(0)) - \beta _{22} S_{2}(0) - \beta _{12} \beta _{21} S_{1}(0) S_{2}(0)\Big \}^{-1}. \end{aligned}$$

Notice that, to the contrary of what happens in the next section for the lockdown case, the value of $\beta _{11}$ for which the denominator is zero does not correspond to a singularity: this is to be expected as it differs from $\beta _{11}^*$ in (1), and we confirmed it numerically.

Taking $\vec I$ as in (13), we get an explicit expression for $\Delta S(\beta _{11})$. For a fixed $\varepsilon$, representing the allowed fractional increment in the number of cases when the school is open, the allowed bound for $\beta _{11}$ is given by

20 $$\begin{aligned} A(\beta _{11})/A(0)\le 1+\varepsilon . \end{aligned}$$

With the parameters used in “There is a phase transition in the effect of school transmission rates on the overall epidemic course during an outbreak (or a variant upsurge)” in "Results" section, this gives the mentioned value $\beta _{11}\le 6.344$.

### SIR for lockdown and its linear approximation

System (9) is suitable to model lockdown as well, provided that the reproduction rate, which is the largest eigenvalue of (12), satisfies $R_0<1$, and that initial conditions taken at time ${\overline{t}}$ have a more substantial number of cases and recovered. A linear approximation of the system is possible as the overall number of active cases is never allowed to grow beyond relatively small fractions of the population, never more than 1% in most countries.

With these conditions, a linear approximation is

21 $$\begin{aligned} {\left\{ \begin{array}{ll} I_{1}'={\overline{\beta }}_{11}S_1({\overline{t}})I_{1}+{\overline{\beta }}_{12}S_1({\overline{t}})I_{2}-I_{1}\\ I_{2}'={\overline{\beta }}_{21}S_2({\overline{t}}) I_{1}+{\overline{\beta }}_{22}S_2({\overline{t}}) I_{2}-I_{2}\\ I_{1}({\overline{t}}), I_{2}({\overline{t}}) >0, \end{array}\right. } \end{aligned}$$

with $$S_1({\overline{t}})+S_2({\overline{t}}) +I_{1}(\overline{t})+I_{2}({\overline{t}})<1$$.

Figure 6 illustrates via a simulation for $S_1({\overline{t}})=0.15<0.2, S_2(\overline{t})=0.7<0.8, I_{1}({\overline{t}})=I_{2}({\overline{t}})=10^{-2}, {\overline{\beta }}_{12}= {\overline{\beta }}_{21} =0.25, {\overline{\beta }}_{22}=1$, and ${\overline{\beta }}_{11}=0,2,4$, the closeness of the linear approximation. The total number of cases simulated from the differential system and the linear approximation are almost indistinguishable in the figure for all values of ${\overline{\beta }}_{11}$; the same holds for each subpopulation.

### Allowed level of school transmission for a successful lockdown

Let us assume that a lockdown is applied from time ${\overline{t}}$ to ${\tilde{t}}$ that successfully eradicates the virus; hence with $I_i({\tilde{t}}) \approx 0$ for $i=1,2$. From the mathematical point of view, we can take the eradication time to be $+\infty$ as the dynamical system reaches an equilibrium with no active cases and does not change afterwards. Hence we consider $\Delta S_i=S_i(\infty )-S_i({\tilde{t}}) \approx S_i({\overline{t}})-S_i(\tilde{t})$. Formulas (16) -(19) apply, with 0 and ${\overline{t}}$ replaced by ${\overline{t}}$ and $\infty$, respectively; the quantities ${\mathcal {I}}_i, \beta _{i,j}$, for $i,j=1,2$, decorated by an overscore; and $I_1(\infty )=I_2(\infty )=0$.

The denominator of (19) with the above changes is singular for

22 $$\begin{aligned} \overline{\beta }_{11}^{*}=\frac{1}{S_{1}({\overline{t}})} -\frac{\overline{\beta }_{12} \overline{\beta }_{21} S_{2}(\overline{t})}{ (1 - \overline{\beta }_{22} S_{2}({\overline{t}}))}. \end{aligned}$$

The numerator at $\overline{\beta }_{11}=\overline{\beta }_{11}^{*}$, on the other hand, is not identically zero; this is seen by substituting the value (22) for $\overline{\beta }_{11}$ in (19), with the adaptations listed above: after some algebra, carried out in Mathematica^TM^, the numerator is seen to equal

23 $$\begin{aligned} \frac{(1 + \overline{\beta }_{21} S_{2}({\overline{t}})) ( \overline{\beta }_{12} I_{2}({\overline{t}}) S_{1}(\overline{t})+I_1({\overline{t}})(1 - \overline{\beta }_{22} S_{2}({\overline{t}})) )}{(1 - \overline{\beta }_{22} S_{2}({\overline{t}}))} \approx -1.6\times 10^{-5}; \end{aligned}$$

the numerical value is computed with the values indicated in “The phase transition is preserved under lock-down, albeit with a different critical point” in "Results" section, namely that

24 $$\begin{aligned} {\overline{\beta }}_{12}= {\overline{\beta }}_{21} =0.25, {\overline{\beta }}_{22}=1, \varepsilon =0.3 \end{aligned}$$

and choosing as initial condition at $t={\bar{t}}=5$ the total number of susceptible and infected obtained from the outbreak scenario, that is

25 $$\begin{aligned} S_1({\overline{t}})\approx 0.1996, S_2({\overline{t}}) \approx 0.7981,I_1({\overline{t}})\approx 1.601\times 10^{-4}, I_2({\overline{t}}) \approx 7.9555\times 10^{-4}. \end{aligned}$$

This indicates that the value in (22) is where the linear approximation breaks down, indicating a transition of phases at $\overline{\beta }_{11}^{*}\approx 4.7630$.

In addition, if we require that school opening does not affect more than a certain percentage the overall incidence proportion by asking that

$$\begin{aligned} A(\overline{\beta }_{11})\le (1+\varepsilon ) A(0) \end{aligned}$$

for some $\varepsilon >0$, then after some algebra, carried out in Mathematica^TM^,we get that

26 $$\begin{aligned} \begin{aligned} \overline{\beta }_{11}\le F(\varepsilon )&= \Big [\varepsilon \Big (-1 + \overline{\beta }_{22} S_2(\overline{t}) + \overline{\beta }_{12} \overline{\beta }_{21} S_1(\overline{t}) S_2(\overline{t})\Big ) \Big (\overline{\beta }_{12}I_2(\overline{t}) S_1(\overline{t}) \\&\quad + \overline{\beta }_{21} I_1(\overline{t}) S_2(\overline{t}) + \overline{\beta }_{22} I_2(\overline{t}) S_2(\overline{t}) + \overline{\beta }_{12} \overline{\beta }_{21} I_1(\overline{t}) S_1(\overline{t}) S_2(\overline{t}) \\&\quad + \overline{\beta }_{12} \overline{\beta }_{21} I_2(\overline{t}) S_1(\overline{t}) S_2(\overline{t})\Big )\Big ]\times \Big \{S_1(\overline{t})\Big [-I_1(\overline{t})- \overline{\beta }_{12}I_2(\overline{t}) S_1(\overline{t})\\&\quad - \varepsilon \overline{\beta }_{12} I_2(\overline{t})S_1(\overline{t}) - \overline{\beta }_{21}I_1(\overline{t})S_2(\overline{t})+ 2 \overline{\beta }_{22} I_1(\overline{t})S_2(\overline{t}) -\varepsilon \overline{\beta }_{21} I_1(\overline{t})S_2(\overline{t})\\&\quad - \varepsilon \overline{\beta }_{22} I_2(\overline{t})S_2(\overline{t}) - \varepsilon \overline{\beta }_{12} \overline{\beta }_{21}I_1(\overline{t}) S_1(\overline{t}) S_2(\overline{t})- \overline{\beta }_{12} \overline{\beta }_{21} I_2(\overline{t}) S_1(\overline{t}) S_2(\overline{t})\\&\quad + \overline{\beta }_{12} \overline{\beta }_{22} I_2(\overline{t})S_1(\overline{t}) S_2(\overline{t})-\varepsilon \overline{\beta }_{12} \overline{\beta }_{21} I_2(\overline{t}) S_1(\overline{t}) S_2(\overline{t}) \\&\quad +\varepsilon \overline{\beta }_{12} \overline{\beta }_{22} I_2(\overline{t})S_1(\overline{t}) S_2(\overline{t}) + \overline{\beta }_{21} \overline{\beta }_{22}I_1(\overline{t}) S_2^2(\overline{t}) - \overline{\beta }^2_{22}I_1(\overline{t}) S^2_2(\overline{t}) \\&\quad + \varepsilon \overline{\beta }_{21} \overline{\beta }_{22} I_1(\overline{t}) S^2_2(\overline{t}) + \varepsilon \overline{\beta }^2_{22} I_2(\overline{t}) S^2_2(\overline{t}) \\&\quad +\varepsilon \overline{\beta }_{12} \overline{\beta }_{21} \overline{\beta }_{22} I_1(\overline{t})S_1(\overline{t})S^2_2(\overline{t}) + \varepsilon \overline{\beta }_{12} \overline{\beta }_{21} \overline{\beta }_{22} I_2(\overline{t}) S_1(\overline{t}) S^2_2(\overline{t})\Big ]\Big \}^{-1} \end{aligned} \end{aligned}$$

With the values as in (24) and (25) we get $F(0.3) \approx 2.9944$.

We compare this expression with the bound in (2), in some numerical examples.

### A complete outbreak-lockdown cycle

A confirmation of the behavior of the effect of school opening on one outbreak-lockdown cycle is shown here via a direct simulation.

Continuing the numerical example of “The phase transition is preserved under lock-down, albeit with a different critical point” in "Results" section, suppose a lockdown is imposed starting from ${\overline{t}}=5$, and a 50% reduction is achieved in the transmission rates different from $\beta _{11}$; Fig. 7 shows that as the outbreak is resolved after the lockdown, the cumulative number of cases is close to that at ${\overline{\beta }}_{11}=0$ when $\beta _{11}$ and ${\overline{\beta }}_{11}$ are close enough to the origin, and sharply deviates otherwise.

### SIR with two subpopulations and vaccination

Adult vaccination can be analyzed with a SVIR model, in which susceptible and recovered of the adult population are vaccinated at a constant rate ${\overline{v}}$^39,40^:

27 $$\begin{aligned} {\left\{ \begin{array}{ll} S_1'=-\widetilde{\beta }_{11}S_1I_1-\widetilde{\beta }_{12}S_1I_2\\ I_1'=\widetilde{\beta }_{11}S_1I_1+\widetilde{\beta }_{12}S_1I_2-I_1\\ R_1'=I_1\\ S_2'=-\widetilde{\beta }_{21}S_2I_1-\widetilde{\beta }_{22}S_2I_2-\overline{v} S_2\\ V_2'=\overline{v} S_2-\widetilde{\beta }^{\sharp }_{22}V_2I_2+\overline{v}R_2\\ I_2'=\widetilde{\beta }_{21}S_2I_1+\widetilde{\beta }_{22}S_2I_2-I_2+\widetilde{\beta }^{\sharp }_{22}V_2I_2\\ R_2'=I_2-\overline{v} R_2, \end{array}\right. } \end{aligned}$$

Here, $V_2$ are the vaccinated in the second population, and $\widetilde{\beta }^{\sharp }_{22}$ is the transmission rate of the virus for vaccinated individuals. According to data related to the efficacy of vaccines, we can approximately take $\widetilde{\beta }^{\sharp }_{22}=0.1\times \widetilde{\beta }_{22}$.

It turns out, however, that we can make a great simplification to (27) in order to ease the mathematical analysis of the upcoming sections. Instead of assuming, as realistic, that individuals, who are vaccinated at rate $\overline{v}$, are still partly susceptible, we can introduce a fictitious smaller vaccination rate *v* which gives complete immunity. Consider, in fact, the system

28 $$\begin{aligned} {\left\{ \begin{array}{ll} S_1'=-\widetilde{\beta }_{11}S_1I_1-\widetilde{\beta }_{12}S_1I_2\\ I_1'=\widetilde{\beta }_{11}S_1I_1+\widetilde{\beta }_{12}S_1I_2-I_1\\ R_1'=I_1\\ S_2'=-\widetilde{\beta }_{21}S_2I_1-\widetilde{\beta }_{22}S_2I_2- v S_2\\ I_2'=\widetilde{\beta }_{21}S_2I_1+\widetilde{\beta }_{22}S_2I_2-I_2\\ R_2'=I_2+ v S_2. \end{array}\right. } \end{aligned}$$

It turns out that if $v=\bar{v}(1-\frac{\widetilde{\beta }^{\sharp }_{22}}{\widetilde{\beta }_{22}})$, and $\widetilde{\beta }^{\sharp }_{22}$ and *I*(*t*) are small (for all *t*), then the curves of infected $I(t)=I_1(t) +I_2(t)$ in (27) and in (28) are very close.

We verify this perhaps slightly counter intuitive statement via simulations for the coupled system, and in a Lemma in Appendix E stated for a single population for simplicity. Simulating the total number of infected *I*(*t*) in (27) and in (28), starting at $t=0$ for simplicity, we see an almost perfect overlap in Fig. 8a. For comparison, we also simulated the case of ${\widetilde{\beta }^{\sharp }_{22}} = 0.5 \times {\widetilde{\beta }_{22}}$ in Fig. 8b, which now shows a substantial divergence.

For these reasons, we adopt from now on model (28) to analyze vaccination.

### A linear approximation to SIR with vaccination

In order to analyze (28) we develop a linearization. Notice that in the linear approximation for the initial phase of an SIR model, the terms $\widetilde{\beta }_{i1}S_iI_1+\widetilde{\beta }_{i2}S_iI_2$ are taken to be zero for both $i=1,2$. With the same assumption in the vaccination case, we get the equation $S_2'=-v S_2$: we therefore use the solution to this equation as linear approximation of $S_2(t)$. This leads to the following linearization

29 $$\begin{aligned} {\left\{ \begin{array}{ll} S_1'=-\widetilde{\beta }_{11}S_1(0) I_1-\widetilde{\beta }_{12}S_1(0)I_2\\ I_1'=\widetilde{\beta }_{11}S_1(0)I_1+\widetilde{\beta }_{12}S_1(0)I_2-I_1\\ R_1'=I_1\\ S_2'=-\widetilde{\beta }_{21}S_2(0) e^{-v t}I_1-\widetilde{\beta }_{22}S_2(0) e^{-v t}I_2-v S_2\\ I_2'=\widetilde{\beta }_{21}S_2(0) e^{-v t}I_1+\widetilde{\beta }_{22}S_2(0) e^{-v t}I_2-I_2\\ R_2'=I_2+v S_2. \end{array}\right. } \end{aligned}$$

From (29) we extract

30 $$\begin{aligned} {\left\{ \begin{array}{ll} I_1'=\widetilde{\beta }_{11}S_1(0)I_1+\widetilde{\beta }_{12}S_1(0)I_2-I_1\\ I_2'=\widetilde{\beta }_{21}S_2(0) e^{-v t}I_1+\widetilde{\beta }_{22}S_2(0) e^{-v t}I_2-I_2. \end{array}\right. } \end{aligned}$$

Figure 9 shows one instance of the effectiveness of the linear approximation with realistic parameters.

### Evidence of a phase transition in $\widetilde{\beta }_{11}$ with critical point $1/{S_1(0)}$ during vaccination

We proceed by using the linear approximation to evaluate the attack rates as function of $\widetilde{\beta }_{11}$; in particular, we focus on the one for the external Population 2. Our calculation is done recursively, as shown in Appendix F. The following theorem summarizes the calculation.

#### Theorem A.4

Assume that $I_1,I_2$ are integrable in $[0,+\infty )$ and let

31 $$\begin{aligned} \widetilde{\mathcal {I}}_1= & {} \widetilde{\mathcal {I}}_1(\widetilde{\beta }_{11})=\int _0^{+\infty }I_1(t) dt, \quad \widetilde{\mathcal {I}}_2=\widetilde{\mathcal {I}}_2(\widetilde{\beta }_{11})=\int _0^{+\infty }I_2(t) dt, \end{aligned}$$

32 $$\begin{aligned} p_{k+1}= & {} I_2(0)+ \frac{\widetilde{\beta }_{21}S_{2}(0) I_1(0)}{(k+1)v-\widetilde{\beta }_{11}S_1(0)+1} \end{aligned}$$

33 $$\begin{aligned} q_{k+1}= & {} \widetilde{\beta }_{22} S_{2}(0)+ \frac{\widetilde{\beta }_{12}\widetilde{\beta }_{21} S_{1}(0) S_{2}(0))}{(k+1)v-\widetilde{\beta }_{11}S_1(0)+1} \end{aligned}$$

for $k=0, 1, \dots$. We have that

34 $$\begin{aligned} \widetilde{\mathcal {I}}_1= & {} \widetilde{\mathcal {I}}_1(\widetilde{\beta }_{11})= \frac{\widetilde{\beta }_{12}S_{1}(0) \widetilde{\mathcal {I}}_2+I_1(0)}{1-\widetilde{\beta }_{11}S_1(0)} \end{aligned}$$

35 $$\begin{aligned} \widetilde{\mathcal {I}}_2= & {} \widetilde{\mathcal {I}}_2(\widetilde{\beta }_{11})= \sum _{i=1}^{\infty } p_i\Bigg (\prod _{r=1}^{i-1} \frac{q_r}{r v+1}\Bigg ) \end{aligned}$$

The proof is in Appendix F, where we also give an explicit expression for $\widetilde{\mathcal {I}}_2$ in terms of hypergeometric functions.

To compute the attack rate for the vaccination case observe that the change in active cases is given by $I_i'$, and the change in recovered cases is $I_i$, $i=1, 2$; the change in infected is then $I'+I$, and the attack rate is

36 $$\begin{aligned} A(\widetilde{\beta }_{11})= & {} \frac{1}{S_1(0)+S_2(0))}\int _0^\infty (I'_1+I_1+I'_2+I_2)dt \nonumber \\= & {} \frac{1}{S_1(0)+S_2(0))}(-I_1(0)+\widetilde{\mathcal {I}}_1-I_2(0)+\widetilde{\mathcal {I}}_2) \end{aligned}$$

The attack rate *A* is divergent as $\widetilde{\beta }_{11}$ approaches $\widetilde{\beta }_{11}^{*}= 1/{S_1(0)}$, see (34), which is an indication that the linear approximation breaks down, and that this value is likely to be the critical point.

In order to contain the increase in the total cases by no more than a proportion $\varepsilon$ we need $\widetilde{\beta }_{11}$ satisfying

37 $$\begin{aligned} A (\widetilde{\beta }_{11})\le A(0) (1+\varepsilon ). \end{aligned}$$

### Estimate of the peak time during vaccination

A further evidence of the critical point is obtained by an estimate of the peak time of the infection from (30). Assuming that the active cases in the two subpopulations peak at approximately the same time ${\overline{t}}$, we set $I_1'(t)=I_2'(t)=0$. The solution is

38 $$\begin{aligned} {\overline{t}}= \frac{1}{v} \log { \frac{-\widetilde{\beta }_{12}S_1(0)\widetilde{\beta }_{21}S_2(0)+ \widetilde{\beta }_{22}S_2(0)(1-\widetilde{\beta }_{11})S_1(0)}{1-\widetilde{\beta }_{11}S_1(0)}}. \end{aligned}$$

Hence, the peak time also diverges at $\widetilde{\beta }_{11}=\widetilde{\beta }_{11}^{*}$.

### Simulations of the phase transition during vaccination

With the realistic values of the parameters used previously,

$$\begin{aligned} S_1(0)=0.198872, S_2(0)=0.794451, I_{1}(0)=1.97281 \times 10^{-5},I_{2}(0)=9.55298 \times 10^{-5} \end{aligned}$$

and

$$\begin{aligned} \widetilde{\beta }_{12}= \widetilde{\beta }_{21} =0.5, \widetilde{\beta }_{22}=2, v=0.1, \end{aligned}$$

we have

$$\begin{aligned} \widetilde{\beta }_{11}^{*}=1/{S_1(0)}=5.02836. \end{aligned}$$

Figure 1 for $t > 18$ illustrates a simulation of the differential system, where it is seen that $\widetilde{\beta }_{11}^{*}=5.02836$ is the critical point for the influence of school opening on the overall epidemic.

To achieve a sensible containment take $\varepsilon =0.3$, in which case (37) gives $\widetilde{\beta }_{11}<3.03111$, a bound also visible in Fig. 1 for $t>18$.

### A SPIAR model

To illustrate how a phase transition mechanism also appears in more elaborate and realistic models, we develop and simulate one example.

We introduce the compartments of susceptibles $S_i$ (not subjected to any virus transmission), presymptomatic $P_i$ (infected in incubation period), asymptomatic $A_i$ (infected not showing symptoms after incubation), infected $I_i$ and recovered $R_i$ for $i=1,2$ corresponding to the two subpopulations. A corresponding system could read as follows:

$$\begin{aligned} \frac{dS_{1}}{dt}= & {}\, - S_1 \left( \beta _{11} (P_1+s A_1)+\beta _{12}(P_2+A_2+\xi I_2)\right) \\ \frac{dP_{1}}{dt}= &{}\, S_1 \left( \beta _{11} (P_1+s A_1)+\beta _{12}(P_2+A_2+\xi I_2)\right) -\kappa P_1 \\ \frac{dI_{1}}{dt}= &{}\, \varepsilon _1\kappa P_1- I_1 \\ \frac{dA_{1}}{dt}= &{}\, (1-\varepsilon _1)\kappa P_1- A_1 \\ \frac{dR_{1}}{dt}= &{}\, (I_1+A_1) \\ \frac{dS_{2}}{dt}= &{} \,- S_2 \left( \beta _{21}(P_1+A_1+\xi I_1)+\beta _{22}(I_2+P_2+A_2) -v\right) \\ \frac{dP_{2}}{dt}= &{} \,S_2 \left( \beta _{21}(P_1+A_1+\xi I_1)+\beta _{22}(I_2+P_2+A_2)\right) -\kappa P_2 \\ \frac{dI_{2}}{dt}= &{} \,\varepsilon _2\kappa P_2- I_2 \\ \frac{dA_{2}}{dt}= &{}\, (1-\varepsilon _2)\kappa P_2- A_2 \\ \frac{dR_{2}}{dt}= & {}\, (I_2+A_2) +v S_2 \end{aligned}$$

where the parameters *s*, $\xi$ represent the fractions of asymptomatic encountered at school and of undetected infected individuals, respectively; $\varepsilon _1$, $\varepsilon _2$ are the fractions of symptomatic in Subclass 1 and Subclass 2, respectively; $\kappa$ the rate of exit from latency period. The recovery rate $\gamma$ is normalized to 1 as before, and $v=0$ if there is no ongoing vaccination.

Parameters have been calibrated as given in Table 1 following standard estimations appearing in literature and data studies^37,41,42^ of 2020 and an estimation of the percentage of asymptomatic and infected people^43^.

**Table 1:** Recap of the model parameters and their selected values for each scenario in SPIAR model.

Parameter	Outbreak	Lockdown	Vaccination
$\beta _{22}$	2	0.5	1.5
$\beta _{12}=\beta _{21}$	0.25	0.1	0.25
*s*	0.9	0.9	0.9
$\xi$	0.3	0.3	0.3
*k*	1	1	1
$\varepsilon _1$	0.75	0.75	0.75
$\varepsilon _2$	0.9	0.9	0.9
*v*	0	0	0.05

Figure 10 shows how phase transitions appear also in the SPIAR model. Here, the active cases are given by the sum $P(t)+I(t)+A(t):=P_1(t)+I_1(t)+A_1(t)+P_2(t)+I_2(t)+A_2(t)$, and three scenarios are considered, as before: outbreak, lockdown, and vaccination. In Fig. 10, the cyan curve corresponds to closed schools, while the green one is a subcritical pattern; the red curves, instead, show the risk that the pandemic spirals out of control because of insufficiently controlled school opening.

### Other case studies

A survey of many detailed, data driven studies related to the effect of school opening during the pandemic shows traces of phases transition in all of them.

España et al. use a detailed compartmental model calibrated on mortality and other estimated and observed data in Bogotà, Colombia, during the whole 2020^17^. The study develops various scenarios of school reopening, and evaluates its impact; the phase transition described in our work appears clearly in Figs. 4 and 5 their: one can see that up to 50% capacity the effect of opening schools is almost negligible, while it becomes substantial above 75% capacity. This leads to the conclusion that there has to be a critical point between these values.

The appearance of a phase transition phenomenon in Rozhnova et al.^12^ has been discussed at length in “Evidence of phase transition appears in several data driven case studies” in "Discussion" section.

Yuan et al. use a detailed compartmental model and data from the second semester 2020 in Toronto, an outbreak context, to estimate the likely impact of school opening, also discussed in “Evidence of phase transition appears in several data driven case studies” section ^16^.

Di Domenico at al. analyse French data for late Spring 2020 in order to predict the effect of various forms of school reopening after the end of the lockdown; the paper only makes predictions for short periods (see their Figs. 3 and 5), and the exact details of the contacts and transmission rates, which are partially estimated and partly obtained from previous measurements, are not provided; still, one can see, especially in their Fig. 5, that the transmission rates are supercritical, and school opening determines a sharp increase in the overall epidemic spreading^11^.

Among the statistical papers, Iwata et al. perform a Time-series analyses using the Bayesian method on Japanese data collected during the initial lockdown, and suggests that school closure did not appear to decrease the incidence of COVID-19^6^. Matzinger et al., on the other hand, use US data from the early stages of the outbreak, and regression analysis; the study finds empirical evidence suggesting that school closings dropped infection rate to half: we can interpret this as a sign that transmission rate in schools was supercritical at that time^10^.

An analysis of data gathered by a surveillance of COVID-19 cases in students and staff after reopening of schools across England showed that in-school infections were much less influential than external ones^7^; a study of Italian data from early Fall 2020, a period of low epidemic incidence, also showed very little transmission taking place in schools^8^. These were typical examples of subcritical in-school transmission rate, probably due to the segmented or very controlled reopening of schools.

## Appendix A: Proof of Lemma A.1

### Proof

Consider, first,

39 $$\begin{aligned} \vec {H}=e^t \vec {I}. \end{aligned}$$

Then

$$\begin{aligned} \frac{d \vec {H}}{dt}= e^{ t}\vec {I}+e^{ t}(A-\text {Id}) \vec {I}=e^{ t}A\vec {I}=A e^{ t}\vec {I}=A \vec {H} \end{aligned}$$

with $$\vec {H}(0)=\vec {I}(0):=\vec {I}_0$$. Since

$$\begin{aligned} \vec {H}=e^{A t} \vec {H}_0=e^{At}\vec {I}_0 \end{aligned}$$

we get that

40 $$\begin{aligned} \vec {I}=e^{-t}e^{At}\vec {I}_0. \end{aligned}$$

We, now, show that

41 $$\begin{aligned} \vec {H}(t)=e^{\lambda _{\text {max}}t}\vec {W}+e^{\lambda _{\text {min}}t}\vec {V} \end{aligned}$$

where $\vec {W}>0$. Then, the assertion of the theorem, which is related to $\vec {I}$, follows immediately from (40).

The rate of growth in the initial exponential phase depends on the largest eigenvalue of the reproduction matrix (12). The result is an immediate consequence of the Perron Frobenius theorem. In fact, since all the elements of *A* are positive, then the eigenvector $\vec {\xi }$ associated to $\lambda _{\text {max}}$ has positive components while the eigenvector $\vec {\eta }$ associated to $\lambda _{\text {min}}$ has at least one negative component. Since

$$\begin{aligned} \vec {H}(t)=e^{\lambda _{\text {max}}t} \begin{bmatrix} \frac{1}{\alpha }(\eta _2\xi _1 I_{1}(0)-\eta _1\xi _1 I_{2}(0))\\ \frac{1}{\alpha }(\eta _2\xi _2 I_{1}(0)-\eta _1\xi _2 I_{2}(0))\\ \end{bmatrix} + e^{\lambda _{\text {min}}t} \begin{bmatrix} \frac{1}{\alpha }(-\eta _1\xi _2 I_{1}(0)+\eta _1\xi _1 I_{2}(0))\\ \frac{1}{\alpha }(-\eta _2\xi _2 I_{1}(0)+\eta _2\xi _1 I_{2}(0))\\ \end{bmatrix} \end{aligned}$$

where

$$\begin{aligned} \alpha =\text {det} \begin{bmatrix} \xi _1 &{} \eta _1 \\ \xi _2 &{} \eta _2 \end{bmatrix} =\xi _1\eta _2-\xi _2\eta _1. \end{aligned}$$

Then, if, for example, $\eta _1<0$ we have $\eta _2\ge 0$ hence $\alpha >0$, that is

$$\begin{aligned} \begin{aligned} W_1&=\frac{1}{\alpha }(\eta _2\xi _1I_{1,0}-\eta _1\xi _1I_{2,0})>0\\ W_2&=\frac{1}{\alpha }(\eta _2\xi _2 I_{1,0}-\eta _1\xi _2I_{2,0})>0.\\ \end{aligned} \end{aligned}$$

Analogously if $\eta _1\ge 0$, then $\eta _2<0$, hence $\alpha <0$ and again $W_1>0$ and $W_2>0$. $\square$

## Appendix B: Proof of Theorem A.2

### Proof

Note first that

$$\begin{aligned} \begin{aligned} \lambda _{max,min}=\frac{\text {tr}(A)\pm \sqrt{(\text {tr}(A))^2-4\text {det}(A)}}{2}&=\frac{a_{11}+a_{22}\pm \sqrt{(a_{11}-a_{22})^2+4a_{12}a_{21}}}{2}\\&> \frac{a_{11}+a_{22}\pm (a_{11}-a_{22})}{2}> 0, \end{aligned} \end{aligned}$$

since all the entries of the matrix *A* are positive. Therefore $\lambda _{max},\lambda _{min}>0$. Furthermore

$$\begin{aligned} \lambda '(a_{11})=\frac{1}{2}\Bigg ( 1+\frac{a_{11}-a_{22}}{\sqrt{(a_{11}-a_{22})^2+4a_{21}a_{12}}}\Bigg )>0 \end{aligned}$$

and

$$\begin{aligned} \lambda ''(a_{11})=\frac{2 a_{12}a_{21}}{((a_{11}-a_{22})^2+4a_{21}a_{12})^{3/2}}>0. \end{aligned}$$

We have

$$\begin{aligned} \lambda '_{max}(0)=\frac{1}{2}\Bigg (1-\frac{a_{22}}{\sqrt{a_{22}^2+4a_{12}a_{21}}} \Bigg ) \end{aligned}$$

and

$$\begin{aligned} \begin{aligned} \lambda '_{max}(a_{22}-\alpha \sqrt{a_{12}a_{21}})&=\frac{1}{2}\Bigg (1-\frac{\alpha \sqrt{a_{12}a_{21}}}{\sqrt{\alpha ^2a_{12}a_{21}+4a_{12}a_{21}}} \Bigg )\\&=\frac{1}{2}\Bigg (1-\frac{\alpha \sqrt{a_{12}a_{21}}}{\sqrt{\alpha ^2+4}\sqrt{a_{12}a_{21}}} \Bigg ) =\frac{1}{2}\Bigg (1-\frac{\alpha }{\sqrt{\alpha ^2+4}}\Bigg ). \end{aligned} \end{aligned}$$

Analogously

$$\begin{aligned} \lambda '_{max}(a_{22}+\alpha \sqrt{a_{12}a_{21}})=\frac{1}{2}\Bigg (1+\frac{\alpha }{\sqrt{\alpha ^2+4}}\Bigg ). \end{aligned}$$

Finally

$$\begin{aligned} \lambda '_{max}(2a_{22})=\frac{1}{2}\Bigg (1+\frac{a_{22}}{\sqrt{a_{22}^2+4a_{12}a_{21}}}\Bigg ). \end{aligned}$$

Observe now that

$$\begin{aligned} \lambda _{{max}}^{\prime } \left( {a_{{22}} - \alpha \sqrt {a_{{12}} a_{{21}} } } \right) - \lambda _{{max}}^{\prime } (0) = & \frac{1}{2}\left( {1 - \frac{\alpha }{{\sqrt {\alpha ^{2} + 4} }}} \right) - \frac{1}{2}\left( {1 - \frac{{a_{{22}} }}{{\sqrt {a_{{22}}^{2} + 4a_{{12}} a_{{21}} } }}} \right) \\ & \quad - \frac{\alpha }{{2\sqrt {\alpha ^{2} + 4} }} + \frac{{a_{{22}} }}{{2\sqrt {a_{{22}}^{2} + 4a_{{12}} a_{{21}} } }} \\ = & \lambda _{{max}}^{\prime } (2a_{{22}} ) - \lambda _{{max}}^{\prime } \left( {a_{{22}} + \alpha \sqrt {a_{{12}} a_{{21}} } } \right). \\ \end{aligned}$$

Also note that

$$\begin{aligned} \lambda '_{max}(a_{22}+\alpha \sqrt{a_{12}a_{21}})-\lambda '_{max}(a_{22}-\alpha \sqrt{a_{12}a_{21}})=\frac{\alpha }{\sqrt{4+\alpha ^2}}. \end{aligned}$$

Then

$$\begin{aligned} \begin{aligned}{}&\frac{\lambda '_{max}(a_{22}+\alpha \sqrt{a_{12}a_{21}})-\lambda '_{max}(a_{22}-\alpha \sqrt{a_{12}a_{21}})}{\lambda '_{max}(a_{22}-\alpha \sqrt{a_{12}a_{21}})-\lambda '_{max}(0)}\\&=\frac{\lambda '_{max}(a_{22}+\alpha \sqrt{a_{12}a_{21}})-\lambda '_{max}(a_{22}-\alpha \sqrt{a_{12}a_{21}})}{\lambda '_{max}(2a_{22})- \lambda '_{max}(a_{22}+\alpha \sqrt{a_{12}a_{21}})} =\frac{\frac{\alpha }{\sqrt{4+\alpha ^2}}}{-\frac{\alpha }{2\sqrt{4+\alpha ^2}}+\frac{a_{22}}{2\sqrt{a_{22}^2+4a_{12}a_{21}}}}\\&=\frac{2\alpha \sqrt{a_{22}^2+4a_{12}a_{21}}}{a_{22}\sqrt{\alpha ^2+4}-\alpha \sqrt{a_{22}^2+4a_{12}a_{21}}}\ge \frac{2\alpha }{\sqrt{\alpha ^2+4}-\alpha }. \end{aligned} \end{aligned}$$

Since $$\sqrt{a_{22}^2+4a_{12}a_{21}}> a_{22}$$ and

$$\begin{aligned} a_{22}\sqrt{\alpha ^2+4}-\alpha \sqrt{a_{22}^2+4a_{12}a_{21}}\le a_{22}\sqrt{\alpha ^2+4}-\alpha a_{22} \end{aligned}$$

and the claim follows. $\square$

## Appendix C: Proof of Corollary A.3

### Proof

We show the result for $\vec {H}$, which is defined in (39). Then, the result for $\vec {I}$ follows straightforwardly using (40).

Note that $\lambda _2>\lambda _1>\lambda _0$ and $\tilde{\lambda _2}> \tilde{\lambda _1}> \tilde{\lambda _0}$. We proceed componentwise. Since $V^0_1>0, V^2_1>0$,

$$\begin{aligned} \begin{aligned} |{H}_1^{\lambda _2}(t)-{H}_1^{\lambda _0}(t)|&=|e^{\lambda _2 t}W^2_1+e^{\widetilde{\lambda _2}t}V^2_1-e^{\lambda _0 t}W^0_1-e^{\widetilde{\lambda _0}t}V^0_1|\\&>e^{\lambda _2 t}W^2_1 \Bigg |1-\frac{W^0_1}{W^2_1}e^{(\lambda _0-\lambda _2)t}-\frac{V^0_1}{W^2_1}e^{(\widetilde{\lambda _0}-\lambda _2)t}\Bigg | \end{aligned} \end{aligned}$$

and noticing that

$$\begin{aligned} \begin{aligned} \lambda _0-\lambda _2&<-\sqrt{2}\sqrt{a_{21}a_{12}}\\ \widetilde{\lambda _0}-\lambda _2&<\widetilde{\lambda _2}-\lambda _2=-2\sqrt{2}\sqrt{a_{21}a_{12}} \end{aligned} \end{aligned}$$

we get

$$\begin{aligned} |{H}^{\lambda _2}_1(t)-{H}^{\lambda _0}_1(t)|>e^{\lambda _2 t}W^2_1 \Bigg |1-\bigg (\frac{W^0_1}{W^2_1}+\frac{V^0_1}{W^2_1}\bigg ) e^{-\sqrt{2a_{12}a_{21}}t}\Bigg | \end{aligned}$$

and setting $\frac{W^0_1+V^0_1}{W^2_1}=:Q_1$ we can pick up *t* such that $1-Q_1e^{-\sqrt{2a_{12}a_{21}}t}>\frac{1}{2}$ that is $e^{\sqrt{2a_{12}a_{21}}t}>2 Q_1$ hence $t>\frac{1}{\sqrt{2a_{12}a_{21}}}\ln (2Q_1)=:t^0_1$ so that finally

42 $$\begin{aligned} |{H}^{\lambda _2}_1(t)-{H}^{\lambda _0}_1(t)|>\frac{1}{2}W_1e^{\lambda _2t}. \end{aligned}$$

On the other hand

43 $$\begin{aligned} \begin{aligned} k|H^{\lambda _1}_1(t)-H^{\lambda _0}_1(t)|&=k|e^{\lambda _1 t}W^1_1+e^{\widetilde{\lambda _1}t}V^1_1-e^{\lambda _0 t}W^0_1-e^{\widetilde{\lambda _0}t}V^0_1|\\&\le k e^{\lambda _1t}W^1_1\bigg (1+e^{(\widetilde{\lambda _1}-\lambda _1)}\frac{V^1_1}{W^1_1}+e^{(\lambda _0-\lambda _1)t}\frac{W^0_1}{W^1_1}+e^{(\widetilde{\lambda _0}-\lambda _1)t}\frac{V^0_1}{W^1_1}\bigg ). \end{aligned} \end{aligned}$$

Using the fact that $\widetilde{\lambda _1}-\lambda _1<0$, $\lambda _0-\lambda _1<0$ and $\widetilde{\lambda _0}-\lambda _1<\widetilde{\lambda _1}-\lambda _1<0$, from (43) we get

44 $$\begin{aligned} k|H^{\lambda _1}_1(t)-H^{\lambda _0}_1(t)| \le k e^{\lambda _1t}W^1_1\bigg (1+\frac{V^1_1+W^0_1+V^0_1}{W^1_1}\bigg )=k W_1 e^{\lambda _1 t}\bigg (1+Q_1+\frac{V^1_1}{W^1_1}\bigg ). \end{aligned}$$

Hence, by (42) and (44) we then get

$$\begin{aligned} \frac{1}{2}W_1e^{\lambda _2t}> k W_1 e^{\lambda _1 t}\bigg (1+Q_1+\frac{V^1_1}{W^1_1}\bigg ) \end{aligned}$$

that is

$$\begin{aligned} e^{(\lambda _2-\lambda _1)t}>2k\bigg (1+Q_1+\frac{V^1_1}{W^1_1}\bigg ) \\ e^{2\sqrt{a_{12}a_{21}}t}>2k\bigg (1+Q_1+\frac{V^1_1}{W^1_1}\bigg ) \end{aligned}$$

hence

$$\begin{aligned} t>\frac{1}{2\sqrt{a_{12}a_{21}}}\ln \bigg (2k\bigg (1+Q_1+\frac{V^1_1}{W^1_1}\bigg )\bigg )=:t^1_1. \end{aligned}$$

Therefore, for $$t\ge \text {max}(t^0_1,t^1_1)$$

$$\begin{aligned} |H^{\lambda _2}_1(t)-H^{\lambda _0}_1(t)|>k|H^{\lambda _1}_1(t)-H^{\lambda _0}_1(t)|. \end{aligned}$$

Analogously one can show that

$$\begin{aligned} |H^{\lambda _2}_2(t)-H^{\lambda _0}_2(t)|\ge k|H^{\lambda _1}_2(t)-H^{\lambda _0}_2(t)| \end{aligned}$$

for $$t\ge \text {max}(t^0_2,t^1_2)$$, where

$$\begin{aligned} \begin{aligned} t^0_2&=\frac{1}{\sqrt{2a_{21}a_{12}}}\ln (2Q_2),\\ Q_2&=\frac{W^0_2+V^0_2}{W^2_2},\\ t^1_2&=\frac{1}{2\sqrt{a_{12}a_{21}}}\ln \bigg (2k\bigg (1+Q_2+\frac{V^2_2}{W^2_2}\bigg )\bigg ). \end{aligned} \end{aligned}$$

So picking up $t>\text {max}(t^0_1,t_1^1,t^0_2,t^1_2)$ and using (40), the claim follows. $\square$

## Appendix D: An application of Theorem A.2

Inequality (14) indicates a *phase transition*. In fact from now on let $a_{22}^2>>a_{12}a_{21}$ and $\alpha =2$. In this case

$$\begin{aligned} \frac{2\alpha }{\sqrt{\alpha ^2+4}-\alpha }\simeq 4.8 \end{aligned}$$

and so by (14) we have

$$\begin{aligned} \Delta _1\ge 4.8 \Delta _0=4.8 \Delta _2. \end{aligned}$$

Given

$$\begin{aligned} \lambda '_{max}(0)=\frac{1}{2}\Bigg (1-\frac{a_{22}}{\sqrt{a_{22}^2+4a_{12}a_{21}}}\Bigg ) \end{aligned}$$

and

$$\begin{aligned} \lambda '_{max}(a_{22}-\alpha \sqrt{a_{12}a_{21}})=\frac{1}{2}\Bigg (1-\frac{\alpha }{\sqrt{\alpha ^2+4}} \Bigg ), \end{aligned}$$

if $\lambda '_{max}(0)<<\lambda '_{max}(a_{22}-\alpha \sqrt{a_{12}a_{21}})/4.8$ then from (14)

45 $$\begin{aligned} \begin{aligned} \lambda '_{max}(a_{22}+\alpha \sqrt{a_{12}a_{21}})&\ge 5.8 \lambda '_{max}(a_{22}-\alpha \sqrt{a_{12}a_{21}})-4.8 \lambda '_{max}(0)\\&\ge 4.8 \lambda '_{max}(a_{22}-\alpha \sqrt{a_{12}a_{21}}). \end{aligned} \end{aligned}$$

Hence, by (45) and since $\lambda '_{max}$ is increasing

$$\begin{aligned} \begin{aligned} 4.8\Bigg (\lambda _{max}(a_{22}-\alpha \sqrt{a_{12}a_{21}})-\lambda _{max}(0)\Bigg )&\le 4.8 \lambda '_{max}(a_{22}-\alpha \sqrt{a_{21}a_{12}})(a_{22}-\alpha \sqrt{a_{12}a_{21}})\\&\le \lambda '_{max}(a_{22}+\alpha \sqrt{a_{12}a_{21}}) (2a_{22}-(a_{22}+\alpha \sqrt{a_{12}a_{21}}))\\&\le \lambda _{max}(2a_{22})-\lambda _{max}(a_{22}+\alpha \sqrt{a_{21}a_{12}}). \end{aligned} \end{aligned}$$

Therefore

$$\begin{aligned} \lambda _{max}(2a_{22})-\lambda _{max}(0)\ge 4.8(\lambda _{max}(a_{22}-\alpha \sqrt{a_{21}a_{12}})-\lambda _{max}(0)). \end{aligned}$$

Hence $\forall 0<a_{11}\le a_{22}-\alpha \sqrt{a_{12}a_{21}}$, we have

$$\begin{aligned} \lambda _{max}(2a_{22})-\lambda _{max}(0)\ge 4.8(\lambda _{max}(a_{11})-\lambda _{max}(0)). \end{aligned}$$

Last inequality tells us that closing schools starting from a reproduction number $2a_{22}$ is much more effective than starting from a reproduction number around $a_{22}$.

## Appendix E: Substitution of susceptible vaccinated with unsusceptible

To give a mathematical justification of our statement consider the two systems (28) and (27) in the case of a single population. Hence, we will compare the two systems

46 $$\begin{aligned} {\left\{ \begin{array}{ll} {\hat{S}}'=-\widetilde{\beta }_{22}{\hat{S}}{\hat{I}}-\overline{v} {\hat{S}}\\ {\hat{V}}'=\overline{v}{\hat{S}}-\widetilde{\beta }^{\sharp }_{22}{\hat{I}}{\hat{V}}\\ {\hat{I}}'=\widetilde{\beta }_{22}{\hat{S}}{\hat{I}}-{\hat{I}}+\widetilde{\beta }^{\sharp }_{22}{\hat{I}}{\hat{V}}\\ {\hat{R}}'={\hat{I}}\\ {\hat{S}}(0)={\hat{S}}_0,{\hat{V}}(0)=0, {\hat{I}}(0)={\hat{I}}_0,{\hat{R}}(0)={\hat{R}}_0. \end{array}\right. } \end{aligned}$$

and

47 $$\begin{aligned} {\left\{ \begin{array}{ll} S'=-\widetilde{\beta }_{22}SI- v S\\ I'=\widetilde{\beta }_{22}SI-I\\ R'=I+ v S\\ S(0)=S_0,I(0)=I_0,R(0)=R_0. \end{array}\right. } \end{aligned}$$

### Lemma F.1

If $v=\bar{v}(1-\frac{\widetilde{\beta }^{\sharp }_{22}}{\widetilde{\beta }_{22}})$ and $\widetilde{\beta }^{\sharp }_{22} \int _0^t I(\tau ) d \tau<<1$ then indicating with ${\hat{I}}$ and *I* the infected in the solutions of the two systems respectively, we have that

48 $$\begin{aligned} |{\hat{I}}(t)-I(t)| \end{aligned}$$

is small for all *t*.

### Proof

Consider the auxiliary system

49 $$\begin{aligned} {\left\{ \begin{array}{ll} {S^*}'=-\widetilde{\beta }_{22}S^*I^*-\overline{v} S^*\\ {{V}^*_S}'=\overline{v}\mu S^*- \widetilde{\beta }_{22}I^*V^*_S-\bar{v}(1-\frac{\widetilde{\beta }^{\sharp }_{22}}{\widetilde{\beta }_{22}}) V^*_S\\ {{V}^*_I}'=\overline{v}(1-\mu )S^*+{\bar{v}}(1-\frac{\widetilde{\beta }^{\sharp }_{22}}{\widetilde{\beta }_{22}}) V^*_S\\ {I^*}'=\widetilde{\beta }_{22}S^*I^*+\widetilde{\beta }_{22}I^*V^*_S-I^*\\ {R^*}'=I^*\\ S(0)=S_0,I(0)=I_0,V_S(0)=V_I(0)=0,R(0)=R_0 \end{array}\right. } \end{aligned}$$

where $V_S$ denote the vaccinated susceptible while $V_I$ the vaccinated immune.

It is easy to see that if one takes ${\hat{S}}=S^*$, ${\hat{V}}=V^*_S+V^*_I$, ${\hat{I}}=I^*$ and ${\hat{R}}=R^*$, these variables satisfy (46) with the term $\widetilde{\beta }^{\sharp }_{22}{\hat{I}}{\hat{V}}$ replaced by $\widetilde{\beta }_{22}I^*V^*_S$; as initially $V^*_S \approx \mu (V^*_S+V^*_I) = \mu {\hat{V}}$, the variables ${\hat{S}}=S^*$, ${\hat{V}}=V^*_S+V^*_I$, ${\hat{I}}=I^*$ and ${\hat{R}}=R^*$ satisfy (46) for small *t*, provided that $\mu = \widetilde{\beta }^{\sharp }_{22}/\widetilde{\beta }_{22}$. For later times, the difference between $V^*_S$ and $\mu (V^*_S+V^*_I) = \mu {\hat{V}}$ is only due the term $-\widetilde{\beta }_{22}I^*V^*_S$; then

$$\begin{aligned} |V^*_S - \mu {\hat{V}} | \le \int _{0}^t \widetilde{\beta }_{22}I^*V^*_S d \tau \le \widetilde{\beta }_{22} \mu \max _{0 \le \tau \le t} V^*_S(\tau ) \int _0^t I(\tau ) d \tau \le \widetilde{\beta }_{22} \frac{\widetilde{\beta }^{\sharp }_{22}}{\widetilde{\beta }_{22}} \int _0^t I(\tau ) d \tau , \end{aligned}$$

which is small by assumption.

On the other hand, $S=S^*+V_S^*$, $I=I^*$ and $R=R^* +V_I^*$ is solution of (47) provided that $v=\bar{v}(1-\frac{\widetilde{\beta }^{\sharp }_{22}}{\widetilde{\beta }_{22}})$.

Then (48) follows as $I=I^* \approx {\hat{I}}$. $\square$

## Appendix F: Proof of Theorem A.4

### Proof

Let

50 $$\begin{aligned} \widetilde{\mathcal {I}}_1^{(k)}:= & {} \int _0^{+\infty }I_1(t) e^{-k v t}dt, \end{aligned}$$

51 $$\begin{aligned} \widetilde{\mathcal {I}}_2^{(k)}:= & {} \int _0^{+\infty }I_2(t) e^{-k v t} dt. \end{aligned}$$

Integrating the first equation in (30), we have

52 $$\begin{aligned} -I_1(0) = (\widetilde{\beta }_{11}S_1(0)-1)\widetilde{\mathcal {I}}_1^{(0)}+\widetilde{\beta }_{12}S_1(0) \widetilde{\mathcal {I}}_2^{(0)} \end{aligned}$$

which implies

53 $$\begin{aligned} \widetilde{\mathcal {I}}_1^{(0)}=\frac{\widetilde{\beta }_{12}S_1(0)\widetilde{\mathcal {I}}_2^{(0)}+I_1(0)}{1-\widetilde{\beta }_{11}S_1(0)} > 0\,\,\, \text { if }\,\, \widetilde{\beta }_{11}S_1(0) < 1. \end{aligned}$$

This proves (34) (note that $\widetilde{\mathcal {I}}_1^{(0)}\equiv \widetilde{\mathcal {I}}_1$).

Integrating the second equation in (30) we get

54 $$\begin{aligned} -I_2(0) = \widetilde{\beta }_{21}S_2(0)\widetilde{\mathcal {I}}_1^{(1)} + \widetilde{\beta }_{22}S_2(0)\widetilde{\mathcal {I}}_2^{(1)} - \widetilde{\mathcal {I}}_2^{(0)} \end{aligned}$$

which implies

55 $$\begin{aligned} \widetilde{\mathcal {I}}_2^{(0)} = \widetilde{\mathcal {I}}_2^{(0)}(\widetilde{\mathcal {I}}_1^{(1)},\widetilde{\mathcal {I}}_2^{(1)}) = \widetilde{\beta }_{21}S_2(0)\widetilde{\mathcal {I}}_1^{(1)} + \widetilde{\beta }_{22}S_2(0)\widetilde{\mathcal {I}}_2^{(1)} + I_2(0). \end{aligned}$$

Substituting in (53), we get

56 $$\begin{aligned} \widetilde{\mathcal {I}}_1^{(0)}=\widetilde{\mathcal {I}}_1^{(0)}(\widetilde{\mathcal {I}}_1^{(1)},\widetilde{\mathcal {I}}_2^{(1)})= \frac{\widetilde{\beta }_{12}S_1(0)(\widetilde{\beta }_{21}S_2(0)\widetilde{\mathcal {I}}_1^{(1)} + \widetilde{\beta }_{22}S_2(0)\widetilde{\mathcal {I}}_2^{(1)} + I_2(0))+I_1(0)}{1-\widetilde{\beta }_{11}S_1(0)} \end{aligned}$$

which in turn implies

57 $$\begin{aligned} \begin{aligned} \widetilde{\mathcal {I}}_2^{(0)}&=\widetilde{\mathcal {I}}_2^{(0)}(\widetilde{\mathcal {I}}_2^{(1)})\\&=\widetilde{\beta }_{21}S_2(0)\widetilde{\mathcal {I}}_1^{(1)}+\widetilde{\beta }_{22}S_2(0)\widetilde{\mathcal {I}}_2^{(1)}+I_2(0) \\&=\widetilde{\beta }_{21}S_2(0)\frac{\frac{I_1(0)}{v}+\frac{1}{v}\widetilde{\beta }_{12}S_1(0)\widetilde{\mathcal {I}}_2^{(1)}}{1-\frac{1}{v}(\widetilde{\beta }_{11}S_1(0)-1)}+\widetilde{\beta }_{22}S_2(0)\widetilde{\mathcal {I}}_2^{(1)}+I_2(0) \\&=\frac{\widetilde{\beta }_{21}S_2(0)I_1(0)}{v-(\widetilde{\beta }_{11}S_1(0)-1)}+I_2(0)+\widetilde{\mathcal {I}}_2^{(1)}\left( \widetilde{\beta }_{22}S_2(0)+\frac{\widetilde{\beta }_{21}S_2(0)\widetilde{\beta }_{12}S_1(0)}{v-(\widetilde{\beta }_{11}S_1(0)-1)}\right) \\&= p_1 + \widetilde{\mathcal {I}}_2^{(1)} q_1. \end{aligned} \end{aligned}$$

For each $k \ge 1$, it follows from (30), and integration by parts that

58 $$\begin{aligned} \begin{aligned} \widetilde{\mathcal {I}}_1^{(k)}&= \int ^{+\infty }_0 I_1 e^{-k v t} dt\\&=\frac{I_1(0)}{k v} + \int ^{+\infty }_0(\widetilde{\beta }_{11}S_1(0)I_{1}+\widetilde{\beta }_{12}S_1(0)I_{2}-I_1)\frac{e^{-k vt}}{k v} dt \\&=\frac{I_1(0)}{k v}+\frac{1}{k v}\left( (\widetilde{\beta }_{11}S_1(0)-1)\widetilde{\mathcal {I}}_1^{(k)}+\widetilde{\beta }_{12}S_1(0)\widetilde{\mathcal {I}}_2^{(k)}\right) \end{aligned} \end{aligned}$$

which yields

59 $$\begin{aligned} \begin{aligned} \widetilde{\mathcal {I}}_1^{(k)}(\widetilde{\mathcal {I}}_2^{(k)}) = \frac{I_1(0)+\widetilde{\beta }_{12}S_1(0)\widetilde{\mathcal {I}}_2^{(k)}}{k v-(\widetilde{\beta }_{11}S_1(0)-1)} > 0, \end{aligned} \end{aligned}$$

and

$$\begin{aligned} \begin{aligned} \widetilde{\mathcal {I}}_2^{(k)}&= \int ^{+\infty }_{0} I_2 e^{-k vt} dt =\\&= \frac{I_2(0)}{k v}+\int ^{+\infty }_{0} \left( \widetilde{\beta }_{21}S_1(0)e^{-vt}I_1 + \widetilde{\beta }_{22}S_1(0)e^{- vt}I_2 - I_2\right) \frac{e^{-kvt}}{kv} dt\\&= \frac{1}{kv}\left( I_2(0) +\widetilde{\beta }_{21}S_2(0) \widetilde{\mathcal {I}}_1^{(k+1)} +\widetilde{\beta }_{22}S_2(0)\widetilde{\mathcal {I}}_2^{(k+1)}- \widetilde{\mathcal {I}}_2^{(k)}\right) \end{aligned} \end{aligned}$$

that gives

$$\begin{aligned} \widetilde{\mathcal {I}}_2^{(k)} = \frac{1}{(kv+1)} (I_2(0)+\widetilde{\beta }_{21}S_1(0)\widetilde{\mathcal {I}}_1^{(k+1)}+\widetilde{\beta }_{22}S_2(0)\widetilde{\mathcal {I}}_2^{(k+1)}). \end{aligned}$$

This implies

60 $$\begin{aligned} \begin{aligned} \widetilde{\mathcal {I}}_2^{(k)}(\widetilde{\mathcal {I}}_2^{(k+1)})&=\frac{1}{kv+1}\left( I_2(0)+\widetilde{\beta }_{21}S_2(0)\frac{I_1(0)+\widetilde{\beta }_{12}S_1(0)\widetilde{\mathcal {I}}_2^{(k+1)}}{(k+1)v-\widetilde{\beta }_{11}S_1(0)+1} +\widetilde{\beta }_{22} S_2(0) \widetilde{\mathcal {I}}_2^{(k+1)}\right) \\&=\frac{1}{kv+1} \left( I_2(0)+\frac{\widetilde{\beta }_{21}S_2(0)I_1(0) }{(k+1)v-\widetilde{\beta }_{11}S_1(0)+1} \right. \\&\left. + \widetilde{\mathcal {I}}_2^{(k+1)}\left( \widetilde{\beta }_{22} S_2(0)+ \frac{\widetilde{\beta }_{12}\widetilde{\beta }_{21}S_1(0)S_2(0)}{(k+1)v-\widetilde{\beta }_{11}S_1(0)+1}\right) \right) \\&=\frac{1}{kv+1} (p_{k+1}+ \widetilde{\mathcal {I}}_2^{(k+1)} q_{k+1} ). \end{aligned} \end{aligned}$$

From (57) and (60)

$$\begin{aligned} \begin{aligned} \widetilde{\mathcal {I}}_2^{(0)}&= p_1 + q_1\widetilde{\mathcal {I}}_2^{(1)}\\&= p_1 + q_1 \frac{1}{v+1}(p_2+q_2H_2)\\&=\quad \\&= \sum _{i=1}^{\infty } p_i\left(\prod _{r=1}^{i-1} \frac{q_r}{r v+1}\right). \end{aligned} \end{aligned}$$

This proves (35) (note that $\widetilde{\mathcal {I}}_2^{(0)}\equiv \widetilde{\mathcal {I}}_2$). Using Wolfram Mathematica for symbolic calculations, we provide an explicit expression for $\widetilde{\mathcal {I}}_2^{(0)}(\widetilde{\beta }_{11})$ in terms of special functions

$$\begin{array}{*{20}l} {\{ S_{2} (0)(1 - \tilde{\beta }_{{11}} S_{1} (0) + v)\} ^{{ - 1}} \left\{ {[I_{2} (0)S_{2} (0)(1 - \tilde{\beta }_{{11}} S_{1} (0) + v)]} \right.} \hfill \\ {\quad \quad \quad \times _{2} F_{2} \left( {1,1 + \frac{1}{v} - \frac{{\tilde{\beta }_{{11}} S_{1} (0)}}{v} + \frac{{\tilde{\beta }_{{12}} \beta _{{21}} S_{1} (0)}}{{v\beta _{{22}} }};1 + \frac{1}{v},1 + \frac{1}{v} - \frac{{\tilde{\beta }_{{11}} S_{1} (0)}}{v};\frac{{\tilde{\beta }_{{22}} S_{2} (0)}}{v}} \right)} \hfill \\ {\left. {\quad \quad \quad + \tilde{\beta }_{{21}} I_{1} (0)S_{2}^{2} (0)_{2} F_{2} \left( {1,1 + \frac{1}{v} - \frac{{\tilde{\beta }_{{11}} S_{1} (0)}}{v} + \frac{{\tilde{\beta }_{{12}} \tilde{\beta }_{{21}} S_{1} (0)}}{{v\beta _{{22}} }};1 + \frac{1}{v},2 + \frac{1}{v} - \frac{{\tilde{\beta }_{{11}} S_{1} (0)}}{v};\frac{{\tilde{\beta }_{{22}} S_{2} (0)}}{v}} \right)} \right\}} \hfill \\ \end{array}$$

where $\ _pF_q(\vec {a};\vec {b};z)$ is the generalized hypergeometric function^44^. $\square$
